# Asciminib vs bosutinib in chronic-phase chronic myeloid leukemia previously treated with at least two tyrosine kinase inhibitors: longer-term follow-up of ASCEMBL

**DOI:** 10.1038/s41375-023-01829-9

**Published:** 2023-01-30

**Authors:** Andreas Hochhaus, Delphine Réa, Carla Boquimpani, Yosuke Minami, Jorge E. Cortes, Timothy P. Hughes, Jane F. Apperley, Elza Lomaia, Sergey Voloshin, Anna Turkina, Dong-Wook Kim, Andre Abdo, Laura Maria Fogliatto, Philipp le Coutre, Koji Sasaki, Dennis Dong Hwan Kim, Susanne Saussele, Mario Annunziata, Naeem Chaudhri, Lynette Chee, Valentin García-Gutiérrez, Shruti Kapoor, Alex Allepuz, Sara Quenet, Véronique Bédoucha, Michael J. Mauro

**Affiliations:** 1 Universitätsklinikum Jena, Jena, Germany; 2 Adult Hematology and INSERM CIC1427, Hôpital Saint-Louis, Paris, France; 3 HEMORIO, State Institute of Hematology Arthur de Siquiera Cavalcanti, Rio de Janeiro, Brazil; 4 Oncoclínica Centro de Tratamento Oncológico, Rio de Janeiro, RJ Brazil; 5 National Cancer Center Hospital East, Kashiwa, Japan; 6 Georgia Cancer Center, Augusta, GA USA; 7 South Australian Health and Medical Research Institute and University of Adelaide, Adelaide, SA Australia; 8 Centre for Haematology Imperial College London, London, UK; 9 Almazov National Medical Research Centre, St. Petersburg, Russia; 10 Russian Research Institute of Hematology and Transfusiology, St. Petersburg, Russia; 11 National Medical Research Center for Hematology, Moscow, Russia; 12 Uijeongbu Eulji Medical Center, Geumo-dong, Uijeongbu-si, South Korea; 13 Instituto do Câncer do Estado de São Paulo (ICESPSP), São Paulo, Brazil; 14 Hospital de Clínicas de Porto Alegre (HCPA), Porto Alegre, Brazil; 15 Charité–Universitätsmedizin Berlin, Berlin, Germany; 16 Department of Leukemia, The University of Texas MD Anderson Cancer Center, Houston, TX USA; 17 Princess Margaret Cancer Centre, University Health Network, University of Toronto, Toronto, ON Canada; 18 III. Medizinische Klinik, Medizinische Fakultät Mannheim der Universität Heidelberg, Mannheim, Germany; 19 Division of Hematology, AORN Cardarelli, Naples, Italy; 20 King Faisal Specialist Hospital & Research Center, Riyadh, Kingdom of Saudi Arabia; 21 Peter MacCallum Cancer Center and The Royal Melbourne Hospital, Parkville, VIC Australia; 22 Servicio de Hematología, Hospital Universitario Ramón y Cajal, Instituto Ramón y Cajal de Investigación Sanitaria (IRYCIS), Madrid, Spain; 23 Novartis Pharmaceuticals Corporation, East Hanover, NJ USA; 24 Novartis Pharma AG, Basel, Switzerland; 25 Memorial Sloan Kettering Cancer Center, New York, NY USA

**Keywords:** Chronic myeloid leukaemia, Targeted therapies, Chronic myeloid leukaemia

## Abstract

Asciminib, the first BCR::ABL1 inhibitor that Specifically Targets the ABL Myristoyl Pocket (STAMP), is approved worldwide for the treatment of adults with Philadelphia chromosome–positive chronic myeloid leukemia in chronic phase (CML-CP) treated with ≥2 prior tyrosine kinase inhibitors (TKIs). In ASCEMBL, patients with CML-CP treated with ≥2 prior TKIs were randomized (stratified by baseline major cytogenetic response [MCyR]) 2:1 to asciminib 40 mg twice daily or bosutinib 500 mg once daily. Consistent with previously published primary analysis results, after a median follow-up of 2.3 years, asciminib continued to demonstrate superior efficacy and better safety and tolerability than bosutinib. The major molecular response (MMR) rate at week 96 (key secondary endpoint) was 37.6% with asciminib vs 15.8% with bosutinib; the MMR rate difference between the arms, after adjusting for baseline MCyR, was 21.7% (95% CI, 10.53–32.95; two-sided *p* = 0.001). Fewer grade ≥3 adverse events (AEs) (56.4% vs 68.4%) and AEs leading to treatment discontinuation (7.7% vs 26.3%) occurred with asciminib than with bosutinib. A higher proportion of patients on asciminib than bosutinib remained on treatment and continued to derive benefit over time, supporting asciminib as a standard of care for patients with CML-CP previously treated with ≥2 TKIs.

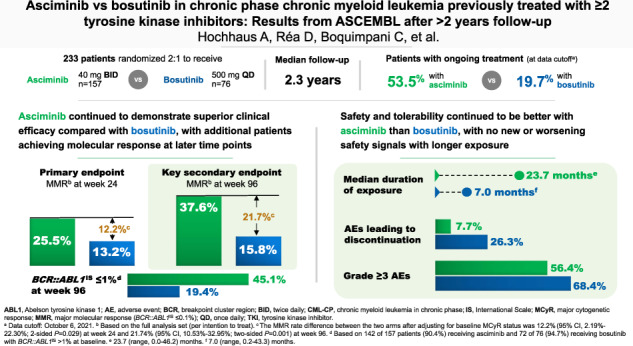

## Introduction

The treatment of patients with chronic myeloid leukemia in chronic phase (CML-CP) after ≥2 prior ATP-competitive tyrosine kinase inhibitors (TKIs) is poorly established owing to a lack of prospective studies in this setting [1–4]. For patients with CML-CP who are intolerant of or resistant to ≥2 prior TKIs, therapeutic options become limited due to newly emerging mutations, existing comorbidities, or toxicities associated with previous treatments [1, 4, 5]. With each subsequent line of TKI treatment, failure rates increase, including the risk of progressing to advanced phases of the disease [6–11]. Patients who require treatment with multiple TKIs may experience severe and/or irreversible adverse events (AEs) due to off-target effects and lack of specificity of ATP-competitive TKIs [1, 12].

Asciminib is the first BCR::ABL1 inhibitor to Specifically Target the ABL Myristoyl Pocket (STAMP) [13]. Based on the results of the pivotal phase 3 study (ASCEMBL) [13] and the phase 1, dose-finding study (NCT02081378) [14**–**16], asciminib was approved in 2021 by the US Food and Drug Administration for the treatment of adults with Philadelphia chromosome–positive CML-CP previously treated with ≥2 TKIs and for those with the *BCR::ABL1* T315I mutation [17], followed by approval in other countries.

Primary (week 24) efficacy and safety results from ASCEMBL in patients with CML-CP after ≥2 prior TKIs were reported previously [13]. Briefly, asciminib demonstrated statistically significant superior efficacy and better safety and tolerability vs bosutinib: the major molecular response (MMR, *BCR::ABL1*^IS^ ≤ 0.1%) rate at week 24 nearly doubled with asciminib compared with bosutinib (25.5% vs 13.2%); the MMR rate difference between arms, adjusted for baseline major cytogenetic response (MCyR), was 12.2% (95% CI, 2.19–22.30%; *p* = 0.029)—meeting the primary objective [13]. Fewer grade ≥3 AEs (50.6% vs 60.5%) and AEs leading to treatment discontinuation (5.8% vs 21.1%) occurred with asciminib than with bosutinib [13].

After 1 year (48 weeks) of follow-up, asciminib continued to show increasing efficacy benefit vs bosutinib, with no new or worsening safety findings as compared with the primary analysis [13, 18]. To further assess the long-term benefits and risks of asciminib compared with those of bosutinib in patients with CML-CP after ≥2 prior TKIs, we report efficacy and safety results in ASCEMBL after a median follow-up of 2.3 years.

## Methods

### Study design and patients

Study design and patient eligibility for ASCEMBL (NCT03106779) were described previously [13]. Briefly, patients (aged ≥18 years) with CML-CP treated with ≥2 prior ATP-competitive TKIs, who experienced treatment failure (lack of efficacy as defined in the 2013 European LeukemiaNet recommendations for second-line TKI therapy [19]) or intolerance of their most recent TKI were randomized 2:1 to receive either asciminib 40 mg twice daily or bosutinib 500 mg once daily, with stratification for MCyR status at baseline (Supplementary Fig. S1). Those harboring T315I or V299L *BCR::ABL1* mutations prior to study entry were ineligible.

Patients meeting lack of efficacy criteria were to permanently discontinue study treatment. A protocol amendment on December 14, 2018, allowed patients for whom bosutinib treatment failed due to lack of efficacy (based on objective laboratory criteria) the possibility to switch to asciminib. Data collected for patients receiving asciminib after switching from bosutinib are not part of this manuscript.

### Procedures

Full details of the primary efficacy and safety assessments were described previously [13]. The key secondary objective was to assess the efficacy of asciminib vs bosutinib at week 96 in patients with CML-CP treated with ≥2 prior TKIs. The key secondary endpoint was the rate of MMR at week 96. To be counted as being in MMR at week 96, patients must have been on study treatment with *BCR::ABL1*^IS^ ≤ 0.1% at week 96 and must not have met any criteria for treatment failure (defined as lack of efficacy per 2013 European LeukemiaNet recommendations or treatment discontinuation for any reason) before this time point. Additional details on secondary and exploratory endpoints and analyses are in the Supplemental Appendix. Analysis sets are in described in Supplementary Table S1.

### Statistical analysis

MMR rate at week 96 was calculated based on the intent-to-treat population (all randomized patients). The Cochran-Mantel-Haenszel chi-square test, stratified by MCyR status at baseline, was used to compare MMR rates between the treatment groups. Hypothesis testing for the primary and key secondary endpoints followed a hierarchical testing approach to preserve an overall alpha level of 5%. Because the test of the primary endpoint was significant, the key secondary endpoint was tested at the 5% level of significance (two-sided test). The Mantel-Haenszel estimates of the common risk difference and the corresponding 95% CIs and the MMR rates and 95% CIs based on the Pearson-Clopper method for each treatment arm are presented.

### Ethics

The study was designed collaboratively by the sponsor and lead study investigators. The protocol was approved by the sites’ institutional review boards and conducted in accordance with the Declaration of Helsinki. All patients provided written informed consent. An independent Data Monitoring Committee reviewed safety data approximately every 6 months. All authors had access to the results and analyzed them in collaboration with the sponsor.

## Results

### Patients

Between November 15, 2017, and December 4, 2019, 233 patients with CML-CP treated with ≥2 prior TKIs were randomized to receive asciminib (*n* = 157) or bosutinib (*n* = 76). Baseline characteristics were reported previously (Supplementary Table S2) [13]. The median follow-up for the current analysis was 2.3 years (120 weeks). The efficacy and safety analyses presented here are based on all data collected up to the cutoff date of October 6, 2021, when all randomized patients had completed their week 96 visit or discontinued earlier.

At the time of data cutoff, study treatment was ongoing in 84 (53.5%) and 15 (19.7%) patients randomized to asciminib and bosutinib, respectively, with 72 (45.9%) and 61 (80.3%) patients having discontinued treatment. One patient assigned to asciminib developed cytopenia after randomization and was not treated per investigator’s decision. As in the primary analysis [13], lack of efficacy (asciminib, 24.2%; bosutinib, 35.5%) remained the most common reason for treatment discontinuation, followed by AEs (asciminib, 7.0%; bosutinib, 25.0%) and physician decision (asciminib, 8.9%; bosutinib, 7.9%) (Table 1).

**Table 1:** Patient disposition.

Variable, *n* (%)	Asciminib 40 mg twice daily (*n* = 157)	Bosutinib 500 mg once daily (*n* = 76)
Patients randomized		
Treated	156 (99.4)	76 (100.0)
Not treated	1 (0.6)^a^	0
Treatment ongoing^b^	84 (53.5)	15 (19.7)
Discontinued treatment	72 (45.9)	61 (80.3)
Before week 24	26 (16.6)	25 (32.9)
Week 24 to before week 48	25 (15.9)	29 (38.2)
Week 48 to before week 96	17 (10.8)	3 (3.9)
At or after week 96	4 (2.5)	4 (5.3)
Reason for discontinuation		
Lack of efficacy	38 (24.2)	27 (35.5)
Adverse event	11 (7.0)	19 (25.0)
Physician decision^c^	14 (8.9)	6 (7.9)
Patient decision	5 (3.2)	4 (5.3)
Death	1 (0.6)	0
Lost to follow-up	1 (0.6)	2 (2.6)
Progressive disease	1 (0.6)	3 (3.9)
Protocol deviation	1 (0.6)	0
Switched to receive asciminib^d^	NA	24 (31.6)

*ELN* European LeukemiaNet, *NA* not applicable.

^a^One patient developed cytopenia after randomization and was not treated per investigator’s decision.

^b^Ongoing at the time of data cutoff: October 6, 2021.

^c^Discontinuations based on physicians’ assessment of lack of efficacy, but not meeting lack of efficacy criteria per ELN 2013 recommendations, were reported as physician decision.

^d^Data collected for patients who switched to receive asciminib are not part of this manuscript.

The median duration of exposure by the data cutoff was 23.7 (range, 0.0–46.2) months for asciminib and 7.0 (0.2–43.3) months for bosutinib. The median dose intensity (range) was 79.7 (33–80) mg/day for asciminib and 463.8 (181–566) mg/day for bosutinib.

### Efficacy

**Fig. 1:**
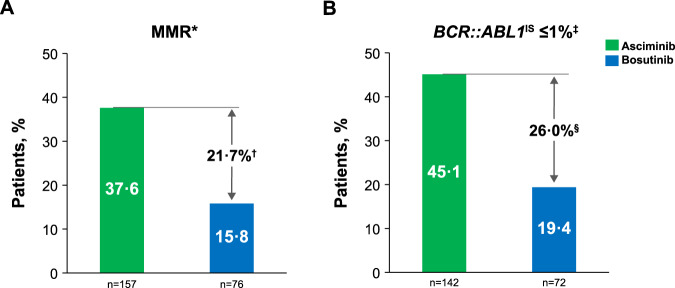
MMR and *BCR::ABL1*^IS^ ≤ 1% at week 96. **A** The MMR graph shows the MMR rates at week 96 for asciminib and bosutinib. **B** The *BCR::ABL1*^IS^ ≤ 1% graph shows the *BCR::ABL1*^IS^ ≤ 1% rate at week 96 for asciminib and bosutinib. MCyR major cytogenetic response, MMR major molecular response (*BCR::ABL1*^IS^ ≤ 0.1% on the International Scale). *Based on the full analysis set. ^†^The MMR rate difference between the two arms after adjusting for baseline MCyR status was 21.74% (95% CI, 10.53–32.95; two-sided *p* = 0.001). ^‡^Based on 142 of 157 (90.4%) patients receiving asciminib and 72 of 76 (94.7%) receiving bosutinib with *BCR::ABL1*^IS^ > 1% at baseline. ^§^The *BCR::ABL1*^IS^ ≤ 1% rate difference between the two arms after adjusting for baseline MCyR status was 26.02% (95% CI, 13.48–38.56; two-sided *p* = 0.000).

**Fig. 2:**
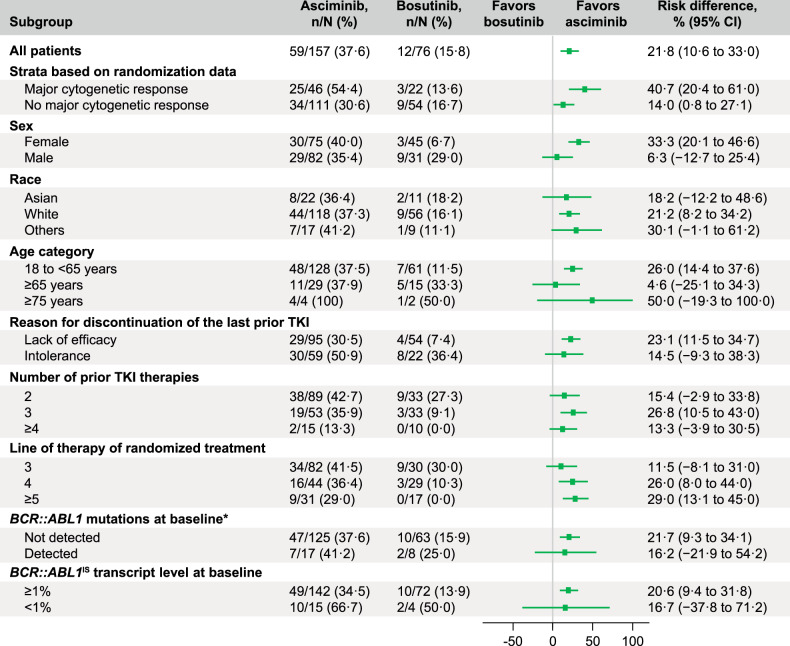
MMR rate difference (95% CI) between treatment at week 96 from subgroup analyses. A forest plot shows the MMR rate difference between treatment arms with 95% CIs at week 96 from subgroup analyses. MMR, major molecular response (*BCR::ABL1*^IS^ ≤ 0.1% on the International Scale); TKI tyrosine kinase inhibitor. *Patients with T315I and V299L *BCR::ABL1* mutations or a non-evaluable mutation assessment were excluded from the subgroup analysis.

**Fig. 3:**
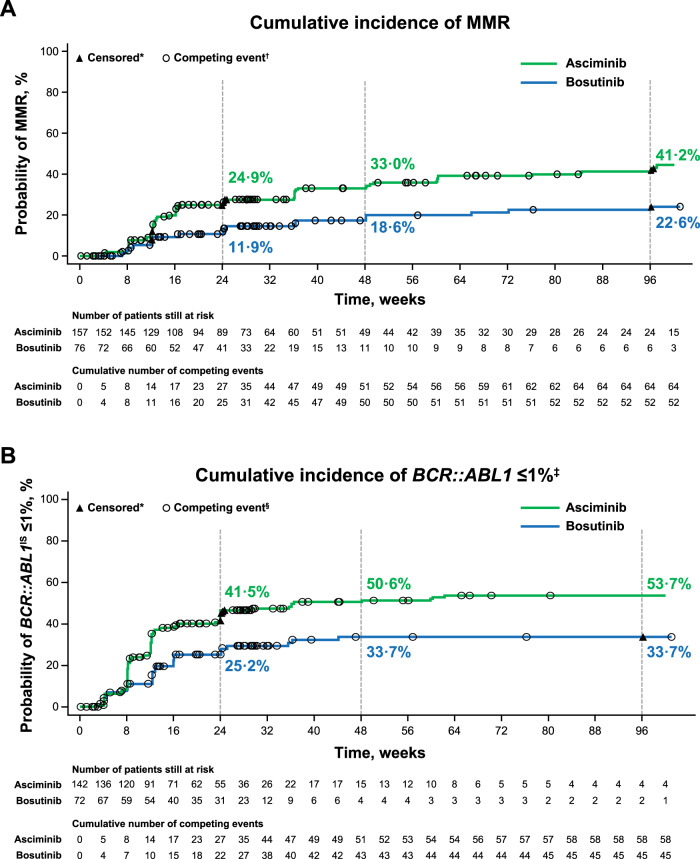
Cumulative incidence of MMR and of *BCR::ABL1*^IS^ ≤ 1%. **A** The cumulative incidence of MMR curve shows the probability of achieving MMR over time in each treatment arm. **B** The cumulative incidence of *BCR::ABL1*^IS^ ≤ 1% curve shows the probability of achieving *BCR::ABL1*^IS^ ≤ 1% over time in each treatment arm. Both were calculated using a competing risk analysis. MMR major molecular response (*BCR::ABL1*^IS^ ≤ 0.1% on the International Scale). *Non-responders were censored at their last molecular assessment date. ^†^Discontinuation from treatment for any reason without prior achievement of MMR is considered a competing event. ^‡^Based on 142 of 157 (90.4%) patients receiving asciminib and 72 of 76 (94.7%) receiving bosutinib with *BCR::ABL1*^IS^ > 1% at baseline. ^§^Discontinuation from treatment for any reason without prior achievement of *BCR::ABL1*^IS^ ≤ 1% is considered a competing event.

The MMR rate at week 96 was 37.6% with asciminib compared with 15.8% with bosutinib (Fig. 1A). The MMR rate difference between the two arms, after adjusting for baseline MCyR status, was 21.74% (95% CI, 10.53–32.95; two-sided *p* = 0.001), meeting the key secondary objective of the study. The MMR rate at week 96 showed a consistent trend in favor of asciminib over bosutinib across all analyzed subgroups for demographic and prognostic factors of response, including baseline MCyR status, reason for discontinuation of the last prior TKI, number of prior lines of TKI therapy, and *BCR::ABL1* mutation status at baseline (Fig. 2, Supplementary Fig. S2). While the cumulative incidence of MMR increased in both arms, the increase was higher with asciminib. MMR was achieved early with asciminib and the difference in cumulative incidence of MMR between arms was evident by week 12. By week 96, the probability of achieving MMR was 41.2% with asciminib and 22.6% with bosutinib (Fig. 3A).

The rate of *BCR::ABL1*^IS^ ≤ 1% at week 96 in patients with *BCR::ABL1*^IS^ > 1% at baseline was 45.1% with asciminib compared with 19.4% with bosutinib (Fig. 1B). Similarly, the cumulative incidence of *BCR::ABL1*^IS^ ≤ 1% by week 96 was higher with asciminib (53.7%) than with bosutinib (33.7%), and differences were evident by week 8. The rate of *BCR::ABL1*^IS^ ≤ 1% for patients receiving asciminib continued to increase after 48 weeks of treatment (Fig. 3B). The rates of MMR and *BCR::ABL1*^IS^ ≤ 1% at and by week 96 are presented in Supplementary Table S3.

MMR and *BCR::ABL1*^IS^ ≤ 1% response levels were durable in both treatment arms. Nearly all patients who achieved MMR (67/69 and 17/18 with asciminib and bosutinib, respectively) or *BCR::ABL1*^IS^ ≤ 1% (74/78 and 23/24 with asciminib and bosutinib, respectively) maintained these levels of response by the time of their last molecular assessment. The probability (95% CI) of maintaining MMR and *BCR::ABL1*^IS^ ≤ 1% for ≥72 weeks was 96.7% (87.4–99.2) and 94.6% (86.2–97.9), respectively, with asciminib and 92.9% (59.1–99.0) and 95.0% (69.5–99.3), respectively, with bosutinib.

At week 96, deep molecular response rates (MR^4^, *BCR::ABL1*^IS^ ≤ 0.01%; and MR^4.5^, *BCR::ABL1*^IS^ ≤ 0.0032%) were consistently higher with asciminib (17.2% and 10.8%, respectively) than with bosutinib (10.5% and 5.3%, respectively) (Supplementary Table S4). The complete cytogenetic response (CCyR) rate at week 96 in patients who were not in CCyR at baseline was 39.8% with asciminib and 16.1% with bosutinib. The CCyR rate difference between the two arms, after adjusting for baseline MCyR status, was 23.9% (95% CI, 10.3–37.4).

By the cutoff date, fewer patients receiving asciminib (51.0%) than bosutinib (82.9%) experienced treatment failure (Supplementary Fig. S3). The median time to treatment failure was much longer with asciminib (24 months) than with bosutinib (6 months).

The 2-year estimated progression-free survival rate (95% CI) was 94.4% (88.6–97.3) with asciminib and 91.1% (79.5–96.3) with bosutinib; the 2-year estimated overall survival rate (95% CI) was 97.3% (92.9–99.0) with asciminib and 98.6% (90.2–99.8) with bosutinib.

### Mutations

Among the 17 of 20 patients receiving asciminib who had *BCR::ABL1* mutations detected by Sanger sequencing at baseline, 15 had ATP-binding site mutations, one had a mutation in the myristoyl pocket region (at a residue not in direct contact with asciminib [20]), and two had mutations in kinase C-terminal or core regions—one of which was combined with an ATP-binding site mutation. Seven of these patients were in MMR at week 96 (six with mutations in the ATP-binding site and one with the mutation in the kinase C-terminal region) (Supplementary Table S5). Among patients who discontinued treatment due to lack of efficacy or disease progression, a similar percentage of patients receiving asciminib (15.4%) and bosutinib (16.7%) had *BCR::ABL1* mutations identified at the end of treatment that were also identified at baseline. The percentage of patients with newly emerging mutations at the end of treatment was 25.6% with asciminib and 6.7% with bosutinib. However, among the patients who discontinued treatment due to lack of efficacy or disease progression, most had no *BCR::ABL1* mutations detected at their end-of-treatment visit in both arms: 56.4% (22/39) with asciminib and 66.7% (20/30) with bosutinib (Supplementary Table S6).

### Safety

Despite the longer duration of exposure on asciminib, a lower proportion of patients receiving asciminib than bosutinib, respectively, experienced all-grade AEs (91.0% vs 97.4%) and grade ≥3 AEs (56.4% vs 68.4%). The most common grade ≥3 AEs (≥10%) with asciminib were thrombocytopenia (22.4%) and neutropenia (18.6%) and with bosutinib were neutropenia (14.5%), diarrhea (10.5%), and increased alanine aminotransferase (ALT) (14.5%) (Table 2). New or worsened post-baseline laboratory abnormalities were mostly grade 1 or 2 (Supplementary Table S7).

**Table 2:** Adverse events regardless of relationship to study drug (reported in ≥5% of patients in any treatment arm).

Event, *n* (%)^a^	Asciminib 40 mg twice daily (*n* = 156)		Bosutinib 500 mg once daily (*n* = 76)	
	All grades	Grade ≥ 3	All grades	Grade ≥ 3
Number of patients with ≥1 adverse event	142 (91.0)	88 (56.4)	74 (97.4)	52 (68.4)
Thrombocytopenia^b^	46 (29.5)	35 (22.4)	15 (19.7)	7 (9.2)
Neutropenia^c^	36 (23.1)	29 (18.6)	16 (21.1)	11 (14.5)
Headache	31 (19.9)	3 (1.9)	12 (15.8)	0
Fatigue	23 (14.7)	1 (0.6)	7 (9.2)	1 (1.3)
Hypertension	21 (13.5)	10 (6.4)	4 (5.3)	3 (3.9)
Arthralgia	20 (12.8)	1 (0.6)	3 (3.9)	0
Diarrhea	20 (12.8)	0	55 (72.4)	8 (10.5)
Nausea	18 (11.5)	1 (0.6)	35 (46.1)	0
Nasopharyngitis	17 (10.9)	0	3 (3.9)	0
Anemia	16 (10.3)	2 (1.3)	6 (7.9)	3 (3.9)
Abdominal pain	14 (9.0)	0	12 (15.8)	1 (1.3)
Pain in extremity	14 (9.0)	1 (0.6)	5 (6.6)	0
Rash	14 (9.0)	0	18 (23.7)	3 (3.9)
Asthenia	13 (8.3)	0	1 (1.3)	0
Cough	13 (8.3)	0	5 (6.6)	0
Back pain	12 (7.7)	1 (0.6)	3 (3.9)	1 (1.3)
Vomiting	12 (7.7)	2 (1.3)	20 (26.3)	0
Dizziness	11 (7.1)	0	2 (2.6)	0
Dyspepsia	11 (7.1)	0	3 (3.9)	0
Insomnia	11 (7.1)	0	1 (1.3)	0
Peripheral edema	11 (7.1)	0	2 (2.6)	0
Upper respiratory tract infection	11 (7.1)	1 (0.6)	4 (5.3)	0
Myalgia	10 (6.4)	0	2 (2.6)	0
Amylase increased	9 (5.8)	1 (0.6)	4 (5.3)	0
Aspartate aminotransferase increased	9 (5.8)	3 (1.9)	16 (21.1)	5 (6.6)
Muscle spasms	9 (5.8)	1 (0.6)	0	0
Constipation	8 (5.1)	0	4 (5.3)	0
Decreased appetite	8 (5.1)	0	6 (7.9)	0
Dry skin	8 (5.1)	0	6 (7.9)	0
Dyspnea	8 (5.1)	0	4 (5.3)	0
Lipase increased	8 (5.1)	6 (3.8)	5 (6.6)	4 (5.3)
Non-cardiac chest pain	8 (5.1)	2 (1.3)	1 (1.3)	0
Oropharyngeal pain	8 (5.1)	0	2 (2.6)	0
Pruritus	8 (5.1)	0	5 (6.6)	1 (1.3)
Rash maculopapular	8 (5.1)	0	2 (2.6)	1 (1.3)
Abdominal pain upper	7 (4.5)	0	5 (6.6)	1 (1.3)
Alanine aminotransferase increased	7 (4.5)	1 (0.6)	23 (30.3)	11 (14.5)
Pyrexia	6 (3.8)	2 (1.3)	6 (7.9)	1 (1.3)
Blood creatinine increased	5 (3.2)	0	5 (6.6)	0
Influenza-like illness	3 (1.9)	0	4 (5.3)	0
Hypophosphatemia	2 (1.3)	1 (0.6)	4 (5.3)	3 (3.9)

^a^Based on the safety analysis set as reported by the investigator. Numbers represent counts of patients. A patient with multiple severity grades for an adverse event is only counted under the maximum grade; Medical Dictionary for Regulatory Activities version 24.1, Common Terminology Criteria for Adverse Events version 4.03.

^b^Includes thrombocytopenia and decreased platelet count.

^c^Includes neutropenia and decreased neutrophil count.

Fewer patients receiving asciminib (7.7%) than bosutinib (26.3%) experienced AEs leading to treatment discontinuation. The most common grade ≥3 AEs leading to treatment discontinuation with asciminib included thrombocytopenia (3.2%) and neutropenia (2.6%) and with bosutinib included increased ALT (3.9%), neutropenia (3.9%), and pleural effusion (2.6%) (Supplementary Table S8). AEs leading to dose adjustment and/or interruption remained lower with asciminib than with bosutinib (42.3% vs 64.5%).

Per protocol, AEs (depending on the type of event and its duration) were managed first by dose interruption and upon resolution, by dose reduction. A lower proportion of patients receiving asciminib than bosutinib had dose modifications of study drug: 66 (42.3%) and 47 (61.8%) patients, respectively, had at least one dose interruption due to AEs; 37 (23.7%) and 34 (44.7%) patients, respectively, had at least one dose reduction due to AEs (Supplementary Table S9).

**Fig. 4:**
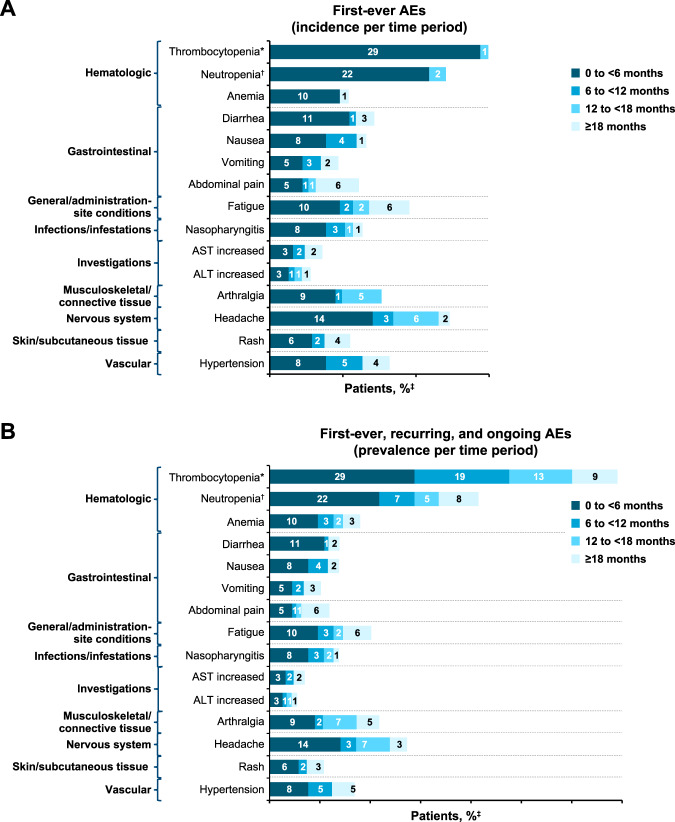
All-grade AEs by time period with asciminib. **A** The first-ever adverse events (AEs) graph shows the incidence of AEs over time. **B** The first-ever, recurring, and ongoing AEs graph shows the prevalence of AEs over time. AE adverse event, ALT alanine aminotransferase, AST aspartate aminotransferase. *Includes thrombocytopenia and platelet count decreased. ^†^Includes neutropenia and neutrophil count decreased. ^‡^A patient with multiple occurrences of an AE is counted only once in that time period. Percentages were rounded to zero decimal places. The denominator for incidence is the number of patients ongoing at the beginning of each time period who have not yet experienced the event. The denominator for prevalence is the number of patients ongoing at the beginning of each time period.

Figure 4 illustrates the incidence and prevalence of all-grade AEs by time period in patients receiving asciminib. Most AEs, both hematologic and non-hematologic, initially presented within the first 6 months of treatment. Few patients experienced a first-ever (incidence) hematologic AE after 6 months, and recurring or ongoing AEs (prevalence) resolved over time. The pattern of non-hematologic AEs over time was similar for incidence and prevalence, with few events persisting or recurring beyond the initial time period of presentation.

The exposure-adjusted incidence rates (EAIRs) for non-hematologic AEs were lower with asciminib than with bosutinib (Supplementary Table S10) and followed a distribution similar to that of the most frequent AEs reported in Table 2. The EAIRs for non-hematologic AEs of special interest (AESI) were also mostly lower with asciminib than with bosutinib (Table 3).

**Table 3:** EAIRs of adverse events of special interest.

EAIR, *n* (per 100 patient-treatment years)^a,b^	Asciminib 40 mg twice daily (*n* = 156)	Bosutinib 500 mg once daily (*n* = 76)
Cardiac failure (clinical events)	3 (1.1)	1 (1.3)
Edema and fluid retention	16 (6.4)	7 (10.1)
Gastrointestinal toxicity	52 (26.6)	60 (319.2)
Hemorrhage	19 (7.4)	8 (11.1)
Hepatotoxicity (including AEs related to laboratory value abnormalities)	17 (6.8)	25 (40.8)
Hypersensitivity^c^	32 (14.0)	26 (48.2)
Pancreatic toxicity	13 (5.1)	7 (10.2)
QTc prolongation	6 (2.3)	1 (1.3)
Reproductive toxicity^d^	3 (1.1)	1 (1.3)

*EAIR* exposure-adjusted incidence rate.

^a^Based on the safety analysis set. Numbers represent counts of patients. Other adverse events of special interest, such as hepatitis B virus reactivation, hepatotoxicity (clinical events), pancreatic toxicity (clinical events), and phototoxicity, were not reported.

^b^Adverse events of special interest are reported as grouped adverse events.

^c^Includes the preferred terms rash, rash maculopapular, dermatitis acneiform, periorbital edema, rash pustular, rhinitis allergic, urticaria, allergic transfusion reaction, dermatitis, dermatitis allergic, dermatitis contact, dermatitis exfoliative generalized, eczema, rash morbilliform, drug eruption, erythema multiforme, eyelid edema, face edema, hand dermatitis, hypersensitivity, and rash erythematous.

^d^Included two events of maternal exposure during pregnancy (with a spontaneous abortion reported in one case) with asciminib and two diagnoses (after informed consent) of congenital cardiovascular anomaly that were not resolved—one each with asciminib and bosutinib.

The frequency of arterial-occlusive events (AOEs) was 5.1% (*n* = 8) with asciminib and 1.3% (*n* = 1) with bosutinib. For asciminib, the EAIR decreased since the primary analysis from 3.3 to 3.0 per 100 patient-years [13] (Supplementary Table S11). Since the primary cutoff, three new patients receiving asciminib experienced AOEs: cerebral infarction, myocardial infarction, and increased troponin (one each). The patient with cerebral infarction previously received nilotinib, dasatinib, and ponatinib and had a history of hypertension and hyperlipidemia. The patient with myocardial infarction previously received imatinib, dasatinib, and nilotinib and had no relevant medical history associated with increased cardiovascular risk. The patient with increased troponin (who reported non-cardiac chest pain on the same day) previously received dasatinib and imatinib. Prior relevant medical conditions for this patient included an implantable defibrillation insertion, and active conditions included myotonic dystrophy, coronary artery disease, decreased ejection fraction, and hyperlipidemia.

Overall, five (3.2%) patients receiving asciminib and two (2.6%) receiving bosutinib died during the study. Deaths occurring by the primary analysis cutoff were previously described [13]. Since the primary cutoff, no additional patients have died on treatment (defined as death occurring during treatment or within 30 days after the end of treatment) and two additional patients died during the survival follow-up (defined as death occurring >30 days after study treatment discontinuation)—one in each treatment arm [13]. One 70-year-old Asian man receiving asciminib for about 9 months had a cerebral infarction during treatment, which led to treatment discontinuation and subsequent death 473 days after the last dose of asciminib. One 46-year-old White woman receiving bosutinib for approximately 6.5 months discontinued because of investigator’s decision. She then underwent a stem cell transplant 12 days after the last dose of bosutinib and received ponatinib treatment for 2 months. She died due to septic shock 1143 days after the last dose of bosutinib.

### Patient-reported outcomes

The completion rate for MD Anderson Symptom Inventory–chronic myeloid leukemia (MDASI-CML) was 96% with asciminib and 92% with bosutinib at baseline and 79.5% and 72.2%, respectively, at week 96. The mean total symptom score at baseline across both treatment arms was 2.0 (on a ten-point scale), with fatigue being the most severe (4.0). Most symptoms either remained stable or decreased in severity from baseline with asciminib (for ≤96 weeks), particularly fatigue, upset feeling, and mood (Supplementary Fig. S4). Many symptoms increased in severity from baseline with bosutinib, most substantially for nausea and diarrhea, the latter being indicative of a clinically meaningful difference in the symptom.

## Discussion

After an additional 16.4 months of follow-up since the ASCEMBL primary analysis, asciminib showed increasingly superior efficacy compared with bosutinib in patients with CML-CP treated with ≥2 prior TKIs—more patients on asciminib achieved clinically relevant and highly durable responses (*BCR::ABL1*^IS^ ≤ 1% and MMR) over time [13].

The MMR rate at week 96 for asciminib was more than double that for bosutinib, meeting the key secondary objective. The statistically significant difference (95% CI) in MMR rates between the two arms increased from 12.2% (2.19–22.30, two-sided *p* = 0.029) at week 24 [13] to 21.74% (10.53–32.95, two-sided *p* = 0.001) at week 96. As in the primary analysis [13], asciminib showed a consistent trend for higher MMR rates at week 96 vs bosutinib across all major demographic and prognostic subgroups analyzed. Asciminib continued to elicit higher MMR rates in both resistant and intolerant patients with greater favorable effect on the resistant cohort; in those with intolerance of their last prior TKI, this effect became more pronounced at week 96 than at week 24 [13], supporting the improved long-term benefit of asciminib. Additionally, the MMR rate at week 96 was also higher in patients on asciminib both with and without any *BCR::ABL1* mutations detected at baseline.

Achieving MMR is associated with a very low risk of disease progression, a high likelihood of subsequent deeper responses, and enhanced survival [21]. However, in heavily pretreated patients, this level of response may be challenging to achieve [9, 10, 14]. For patients treated with ≥2 prior TKIs, a response level of *BCR::ABL1*^IS^ ≤ 1%, or CCyR, is considered advantageous for optimal survival [4].

A sustained clinical benefit was seen with asciminib as demonstrated by the increasing number of patients who achieved *BCR::ABL1*^IS^ ≤ 1% and MMR by weeks 48 and 96 (Fig. 3). The cumulative incidence of *BCR::ABL1*^IS^ ≤ 1% with asciminib increased from 41.5% by week 24 to 53.7% by week 96, with most patients achieving this response by week 48; the cumulative incidence of MMR increased steadily from 24.9% by week 24 to 41.2% by week 96 [13]. These results support *BCR::ABL1*^IS^ ≤ 1% and MMR as attainable treatment response milestones beyond second-line therapy [2] and demonstrate the value of prolonged asciminib treatment.

Encouragingly, deep molecular response rates increased over time and continued to be higher with asciminib than with bosutinib. MR^4^ and MR^4.5^ rates with asciminib at week 96 were comparable to the cumulative rates reported with imatinib in newly diagnosed patients [22], which indicated that deeper responses and treatment-free remission could still be a treatment goal for patients in later lines.

Conclusions on the impact of *BCR::ABL1* mutations on the efficacy of asciminib or on asciminib’s mutational profile cannot be made due to the insufficient number of patients in whom mutations were reported and the diversity of these mutations. No new information regarding the impact of mutations was revealed since the primary analysis [13]. Drug-resistance in CML is recognized as a complex and multifactorial process involving mechanisms other than *BCR::ABL1* mutation–driven resistance, which can operate individually or together [23, 24]. In ASCEMBL, among patients who discontinued treatment due to lack of efficacy or disease progression in both treatment arms, less than half had *BCR::ABL1* mutations detected at the end of treatment (by Sanger sequencing), suggesting alternative mechanisms of resistance may be involved. Newly emerging mutations detected at the end of treatment among patients who discontinued due to lack of efficacy or disease progression were more common with asciminib than with bosutinib (25.6% vs 6.7%). This observation may be an artifact, attributable to the longer duration of treatment with asciminib and higher fraction of bosutinib-treated patients discontinuing treatment early; 25 of the 61 patients who discontinued bosutinib did so before week 24. As noted previously, the frequency of mutations detected at baseline and end of treatment among patients discontinuing due to lack of efficacy or disease progression was similar with asciminib (15.4%) and bosutinib (16.7%); however, the median time to treatment failure was 2 years and 6 months, respectively.

Interpretation of the safety results also warrants consideration that the duration of exposure was much longer for asciminib (approximately three times longer) than for bosutinib by the data cutoff, leading to a longer time for AE reporting. The median duration of exposure for asciminib increased since the primary analysis from 10.0 months to 23.7 months [13]. Despite this, safety and tolerability were consistent and comparable to that at the primary analysis, with no new or worsening safety findings [13].

The burden of AEs with asciminib (both incidence and prevalence) decreased over time in patients who continued treatment beyond 6 months. Consistent with reports on other TKIs [12, 25, 26], most hematologic AEs were observed within the initial months of treatment. Recurring/ongoing hematologic AEs were manageable, and rates of thrombocytopenia (3.2%) and neutropenia (2.6%) leading to discontinuation of asciminib remained low and were similar to those from the primary analysis [13]. Most non-hematologic AEs also presented within the initial months of treatment with low likelihood of recurrence and did not lead to any treatment discontinuation, further indicating manageability of AEs with and tolerability of asciminib.

The decreased burden of AEs over time was consistent with the lower EAIR of AEs with asciminib than with bosutinib. Regardless of the longer duration of exposure for asciminib vs bosutinib, the risk of AEs did not increase, allowing more than half of the patients receiving asciminib (53.5%) to remain on therapy with the continued benefit of deepening responses, which was especially relevant in these hard-to-treat patients who were previously treated with ≥2 TKIs. Fewer patients remained on bosutinib therapy (19.7%), similar to that at the primary cutoff, when 61.8% vs 28.9% of patients were still receiving treatment with asciminib vs bosutinib, respectively [13].

The safety and tolerability of bosutinib in ASCEMBL is comparable to that of bosutinib in the BYOND study [27]. As in ASCEMBL, patients in BYOND received bosutinib at an initial dose of 500 mg once daily [27]. The frequency of discontinuations due to AEs with bosutinib in ASCEMBL was the same as in BYOND (25.0%), despite the median dose intensity being higher in ASCEMBL (463.8 mg/day) than in BYOND (313.1 mg/day) [27]. The median duration of exposure to bosutinib was much lower in ASCEMBL than in BYOND (7.0 months vs 23.7 months); [27] however, this lower exposure cannot be attributed to AEs leading to bosutinib discontinuation, as dose reductions to allow for management of AEs were allowed in ASCEMBL, but rather to the protocol-mandated adherence to the more stringent European LeukemiaNet treatment failure criteria when compared with BYOND. In ASCEMBL, per protocol, patients were to be discontinued from study treatment when lack of efficacy criteria were met [13]. In contrast, in BYOND, patients were to receive bosutinib until disease progression [27]. In ASCEMBL, 35.5% of patients discontinued bosutinib due to lack of efficacy; in BYOND, 5.1% of patients discontinued due to insufficient response [27]. The most common all-grade AEs leading to bosutinib discontinuation were similar in ASCEMBL and BYOND, mostly due to abnormal liver function test results: increased ALT and AST in 5.3% and 2.6% of patients, respectively, in ASCEMBL and in 4.9% and 2.5% of patients in BYOND [27]. Pleural effusion and neutropenia (3.9% each) also led to discontinuations in ASCEMBL (Supplementary Table S8). The frequency of most gastrointestinal AEs leading to bosutinib discontinuation was lower in ASCEMBL than in BYOND (diarrhea, 2.6% vs 1.2%; nausea, 0% vs 1.8%; and vomiting, 0% vs 1.2%) (Supplementary Table S8) [27]. These data support the validity of the results with bosutinib in ASCEMBL, confirming that earlier discontinuations from bosutinib treatment were due to lack of efficacy rather than intolerance.

Overall, the risk of AOEs with asciminib did not increase with longer-term follow-up. The EAIR of AOEs with asciminib decreased from 3.3 per 100-patient years in the primary analysis to 3.0 in the current analysis. The EAIR of AOEs with bosutinib also decreased from 2.0 to 1.4; however, most patients receiving bosutinib discontinued early, thus preventing a meaningful comparison between the two arms. Patients were not stratified by cardiovascular disorders and prior TKI exposure at baseline, hence baseline risk for AOEs was not equivalent between treatment arms. The risk of developing AOEs is seen with most ATP-competitive TKIs, is heightened in patients who have other risk factors for these events, and increases with an increase in comorbidities [28]. In ASCEMBL, AOEs occurred in eight patients with asciminib—most of whom had cardiovascular risk factors and/or were heavily pretreated with ATP-competitive TKIs: six of eight had cardiovascular risk factors at screening, all eight had prior exposure to nilotinib and/or dasatinib, and three had prior ponatinib treatment (Supplementary Table S12). These AOEs do not constitute a safety signal. In accordance with clinical practice guidelines, it is important to closely monitor and manage risk factors and comorbidities during therapy [29].

Overall, after >2 years of follow-up in ASCEMBL, asciminib remained consistently superior to bosutinib with clinically relevant and durable efficacy. Treatment was ongoing in >50% of patients receiving asciminib, allowing for greater potential benefit from long-term asciminib therapy and opportunity to achieve deeper levels of response. These high response rates to a specific inhibitor emphasize that BCR::ABL1 remains the driving force in most patients with CML-CP, even in later lines of therapy, and highlight the need for highly potent, well-tolerated, and targeted TKIs.

Collectively, these updated results from ASCEMBL are a confirmation of the enduring clinical benefit of asciminib after longer exposure and continue to illustrate that asciminib has transformed CML treatment as a new standard of care for patients with CML-CP treated with ≥2 prior TKIs and support its ongoing development in earlier lines of therapy.

## Supplementary information

Supplemental Appendix

Figure S1

Figure S2

Figure S3

Figure S4

Table S1

Table S2

Table S3

Table S4

Table S5

Table S6

Table S7

Table S8

Table S9

Table S10

Table S11

Table S12

CONSORT Checklist

## Data Availability

Novartis is committed to sharing access to patient-level data and supporting clinical documents from eligible clinical trials with qualified external researchers upon request. These requests are reviewed and approved by an independent review panel based on scientific merit. All data provided are anonymized to respect the privacy of patients who have participated in the trial consistent with applicable laws and regulations. The data sets generated during and/or analyzed during the current trial are available according to the criteria and process described on www.clinicalstudydatarequest.com.
