# Supplementary material for: Asciminib vs bosutinib in chronic-phase chronic myeloid leukemia previously treated with at least two tyrosine kinase inhibitors: longer-term follow-up of ASCEMBL
Source: Leukemia. 2023 Jan 30;37(3):617–26. doi: 10.1038/s41375-023-01829-9 (PMC9991909; doi:10.1038/s41375-023-01829-9)
Supplement: Supplementary file 1 — Supplemental Appendix [file 41375_2023_1829_MOESM1_ESM.pdf]

Supplemental Appendix

Table of contents

**Supplemental methods**	2
Patients	2
Study design and treatments	2
Dose adjustments and interruptions	2
Secondary and exploratory endpoints	3
Efficacy analyses	3
Mutational analyses	3
Patient-reported outcomes	3
References for supplemental appendix	5
**Figures**	6
Figure S1: Study schema	6
Figure S2: MMR by line of therapy at week 96	7
Figure S3: Time to treatment failure	8
Figure S4: MDASI-CML MMRM mean change (95% CI) from baseline to week 96: symptom and interference items	9
**Tables**	10
Table S1: Analysis sets	10
Table S2: Demographic and clinical characteristics of patients at baseline	11
Table S3: MMR and *BCR::ABL1*^{IS} ≤1% at and by time points	13
Table S4: MR^4 and MR$^{4.5}$ rates at weeks 24 and 96	15
Table S5: *BCR::ABL1* mutations at baseline and best response by data cutoff	16
Table S6: *BCR::ABL1* mutations at the end of treatment in patients who discontinued treatment	17
Table S7: Laboratory abnormalities	18
Table S8: Adverse events leading to treatment discontinuation of study treatment by preferred term	19
Table S9: Dose adjustments and discontinuation of study drug	20
Table S10: Exposure-adjusted incidence rate of non-hematologic adverse events by preferred term (reported in ≥5% of patients in any treatment arm as shown in Table 2)	21
Table S11: Arterial-occlusive events	23
Table S12: Characteristics of patients with arterial-occlusive events	24

Supplemental methods

Patients

At screening, *BCR::ABL1* transcript levels on the International Scale (*BCR::ABL1*^{IS}) must have been ≥1%. After protocol amendment 3 on December 14, 2018, patients intolerant to their most recent tyrosine kinase inhibitor (TKI) with *BCR::ABL1*^{IS} >0.1% were included. Intolerance was defined as non-hematologic grade 3 or 4 toxicity while on treatment; persistent grade 2 toxicity that is unresponsive to optimal management, including dose adjustments; or hematologic grade 3 or 4 toxicity while on treatment that recurs after dose reduction to the lowest recommended dose. Those with known bosutinib-resistant *BCR::ABL1* mutations of T315I or V299L detected at any time prior to study entry or with a known second occurrence of chronic myeloid leukemia (CML) in chronic phase after previous progression to the accelerated phase (AP) or blast phase (BP), or cardiac disorders or repolarization abnormalities, were excluded.

Study design and treatments

Study design

Patients with treatment failure—defined as meeting lack of efficacy criteria based on the 2013 European Leukemia (ELN) recommendations for second-line TKI therapy—in either treatment arm must be discontinued from study treatment. Following a protocol amendment on December 14, 2018, patients meeting lack of efficacy criteria while receiving bosutinib within 96 weeks after the last patient had been randomized on study had the option to switch to asciminib. Those who switched to asciminib had the opportunity to receive asciminib up to the end of the study treatment period (defined as up to 96 weeks after the last patient receives the first dose or up to 48 weeks after the last patient has switched to asciminib treatment, whichever is longer, unless patients have discontinued treatment earlier). Patients who discontinued bosutinib for any reason other than lack of efficacy (eg, intolerance, disease progression) were not allowed to switch to asciminib.

Patients who discontinue treatment prior to the end of the study period are followed up for progression-free survival and overall survival for ≥5 years from the date at which the last randomized patient received the first dose of study treatment.

Patients must meet at least one of the following criteria to be considered as having lack of efficacy (based on the 2013 ELN recommendations):

- 3 months after initiation of therapy or thereafter: no complete hematologic response or >95% Philadelphia chromosome–positive (Ph+) metaphases
- 6 months after initiation of therapy or thereafter: *BCR-ABL1*^{IS} >10% and/or >65% Ph+ metaphases
- 12 months after initiation of therapy or thereafter: *BCR-ABL1*^{IS} >10% and/or >35% Ph+ metaphases
- At any time after initiation of therapy: loss of complete hematologic response, complete cytogenetic response (CCyR), or partial cytogenetic response
- At any time after initiation of therapy: detection of new *BCR::ABL1* mutations, which potentially cause resistance to study treatment (asciminib or bosutinib)
- At any time after initiation of therapy: confirmed loss of major molecular response (MMR) per two consecutive tests
- At any time after initiation of therapy: new clonal chromosome abnormalities in Ph+ cells

Randomization and masking

Patients were randomized (2:1) to receive asciminib or bosutinib based on a computer-generated randomized list via an interactive web-based response system. The investigator or their designee confirmed if patients fulfilled all eligibility criteria prior to dosing. Each patient was then assigned a randomization number through the system, which linked them to a treatment arm.

Treatments

Asciminib 40 mg twice daily was administered orally without food, and bosutinib 500 mg once daily was administered orally with food.

Dose adjustments and interruptions

For patients who were unable to tolerate the protocol-specified dosing schedule, dose interruptions and/or reductions were either recommended or mandated to allow patients to continue study treatment. For asciminib, only a one-step dose reduction to a total daily dose of 40 mg was allowed. The starting dose level of 40 mg twice daily could be reduced to 20 mg twice daily (dose level − 1). For bosutinib, a two-step sequential dose reduction up to a total daily

dose of 300 mg was allowed. The starting dose level of 500 mg once daily could be reduced to 400 mg once daily (dose level − 1) or 300 mg once daily (dose level − 2).

If after treatment is resumed at a lower dose level the toxicity recurs at the same or worse severity (except for recurrence of cytopenias), the patient must discontinue treatment with either agent. If dose interruption is more than 28 days for each nonhematologic toxicity, the patient must discontinue study treatment. If a hematologic toxicity (cytopenia grade 3 or 4) lasts for more than 42 days without recovery to grade 2 or less, even with dose interruption and adequate management (including hematopoietic growth factors), the patient must discontinue treatment.

Secondary and exploratory endpoints

Other secondary endpoints included MMR, *BCR::ABL1*^{IS} ≤1%, and cytogenetic response rates at and by scheduled time points, cumulative incidence and duration of MMR and *BCR::ABL1*^{IS} ≤1%, time to treatment failure, progression-free survival, overall survival, and safety and tolerability. Patient-reported health-related quality of life was assessed as an exploratory endpoint using the MD Anderson Symptom Inventory–chronic myeloid leukemia (MDASI-CML) questionnaire to compare changes in disease-related symptoms and symptom interference with daily life from baseline.

Time to treatment failure was defined as the time from the date of randomization to an event of treatment failure. Treatment failure was defined as lack of efficacy per the 2013 ELN recommendations for second-line treatment adapted to include discontinuation of randomized treatment as an event. For patients in the full analysis set who had not reached treatment failure, their times to treatment failure were censored at the time of their last study assessment before the cutoff date.

Progression-free survival was defined as the time from the date of randomization to the earliest occurrence of documented disease progression to AP or BP or the date of death from any cause (including progressions and deaths observed during the survival follow-up period) before the data cutoff. For patients who had not experienced an event (disease progression to AP/BP or death from any cause), their Progression-free survival times were censored at the date of their last study assessment before the cutoff date, regardless of subsequent intake of treatments after randomization.

Overall survival was defined as the time from the date of randomization to the date of death (including the survival follow-up period). Patients who were alive at the time of the analysis data cutoff date were censored at the date of their last contact before the cutoff date, regardless of subsequent intake of treatments after randomization.

Efficacy analyses

Cumulative incidence of MMR and *BCR::ABL1*^{IS} ≤1% was analyzed considering discontinuation from treatment for any reason and without prior achievement of MMR/*BCR::ABL1*^{IS} ≤1% as competing risks. Time to MMR/*BCR::ABL1*^{IS} ≤1% was censored at the last molecular assessment date, on treatment, and prior to or at the cutoff date, if no events or competing risks occurred before or at the cutoff date, or at the end of treatment.

Response rates by a given time point were calculated based on the cumulative number of patients who achieved a response at any time up to this time point. Response rates at a given time point were calculated based on the number of patients with a response at this time point, regardless of whether they had previously achieved a response. Cytogenetic response was assessed locally as the percentage of Ph+ metaphases in the bone marrow. The CCyR endpoint was analyzed only in patients who were not in CCyR at baseline. After randomization, bone marrow assessments were required at screening or baseline (performed up to 56 days prior to week 1 day 1); at weeks 24, 48, 72, and 96 only if a patient was not in MMR; and at the end of treatment. If a patient was in MMR at the same time when a bone marrow assessment was scheduled, the CCyR was imputed from the MMR on a specific date if there was no valid cytogenetic assessment.

Mutational analyses

BCR::ABL1 mutational analyses were performed at a central laboratory by Sanger sequencing on day 1 of week 1 and upon confirmed loss of MMR and/or at the end of treatment. Mutational analyses were performed at week 12 and every 12 weeks thereafter up to the end of study treatment, only for patients who had mutations on day 1 of week 1.

Patient-reported outcomes

Patient-reported health-related quality of life was assessed as an exploratory endpoint using the MD Anderson Symptom Inventory–chronic myeloid leukemia questionnaire at screening or baseline and at weeks 4, 8, 12, 16, 24, 36, 48, and 96 while patients remained in the study. Week 96 data are reported in the current analysis. The MD

Anderson Symptom Inventory–chronic myeloid leukemia questionnaire is a 26-item, self-administered questionnaire for adult patients with CML that assesses 20 items relating to severity of disease-related symptoms and six items relating to symptom interference with daily life. Items are scored from zero to ten, with higher scores indicating more severe symptoms and greater interference of symptoms with daily life. The symptom severity score is the mean of the 20 symptom items, and the symptom distress score is the mean of the six interference items.[1] A change in score of 1.5 points was interpreted as a clinically meaningful difference.[2,3] The analysis of the change from baseline was conducted using a mixed-effects model for repeated measures, which adjusts for repeated assessments per patient over time as well as the baseline patient-reported outcome score and covariates. The baseline patient-reported outcome score, stratification factor, treatment arm, study visit, and interaction of treatment arm and study visit were included in the models as fixed effects; patient was included as a repeated effect. An unstructured covariance matrix was used as recommended for repeated-measures models. The completion rate was summarized as a percentage of the number of randomized patients who were expected at that visit; patients expected at each post-baseline visit were those who remained on randomized treatment and had not progressed, switched treatment, died, or withdrew from the study for any other reasons at that visit.

References for Supplemental Appendix

1. Williams LA, Garcia Gonzalez AG, Ault P, et al. Measuring the symptom burden associated with the treatment of chronic myeloid leukemia. *Blood*. 2013; **122**: 641–7.
2. Cleeland C. The M. D. Anderson Symptom Inventory User Guide Version 1. 2016. Accessed November 10, 2022. https://www.mdanderson.org/documents/Departments-and-Divisions/Symptom-Research/MDASI_userguide.pdf
3. Institut für Qualität und Wirtschaftlichkeit im Gesundheitswesen. IQWiG General Methods: Version 6.0 of 5 November 2020. Accessed November 10, 2022. https://www.iqwig.de/methoden/general-methods_version-6-0.pdf

Figures

Figure S1: Study schema

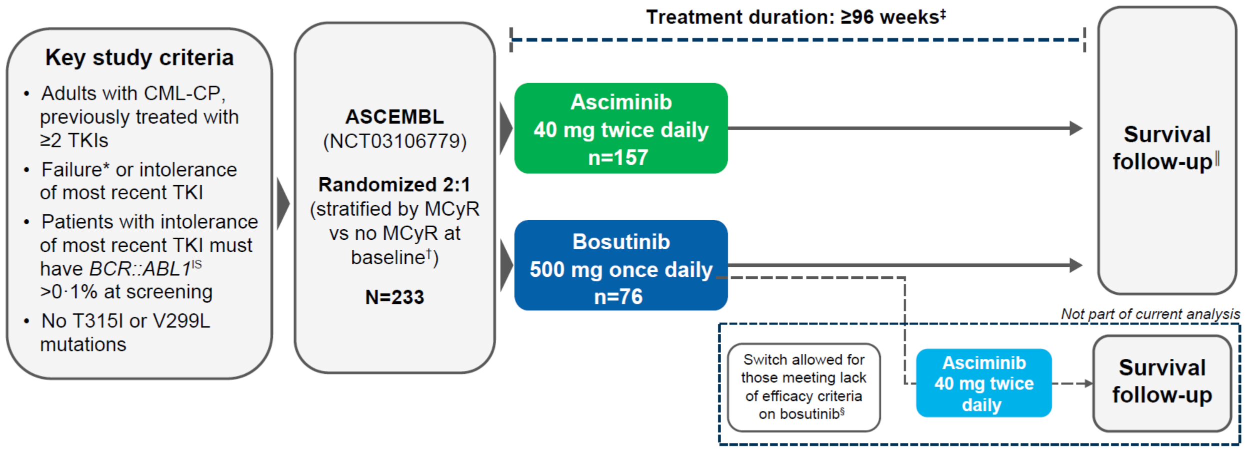

Reprinted from Rea D, et al. *Blood* 2021; **138:** 2031–2041. Copyright © 2021 American Society of Hematology.
2L, second line; CML-CP, chronic myeloid leukemia in chronic phase; MCyR, major cytogenetic response; TKI, tyrosine kinase inhibitor.
* Must meet lack of efficacy criteria based on 2013 ELN recommendations for 2L TKI therapy.
† At baseline, 68 (29.2%) patients had an MCyR and 165 (70.8%) had no MCyR: 46 (29.3%) patients receiving asciminib and 22 (28.9%) receiving bosutinib had an MCyR at baseline.
‡ Patients will continue to receive study treatment for up to 96 weeks after the last patient's first dose or 48 weeks after the last patient switches to asciminib, whichever is longer.
§ Patients who discontinue bosutinib treatment due to intolerance or any reason other than lack of efficacy are not allowed to switch to asciminib.
‖ Patients who discontinue study treatment at any time will be followed-up for survival and progression to AP/BP for up to 5 years after the last patient's first dose.

Figure S2: MMR by line of therapy at week 96

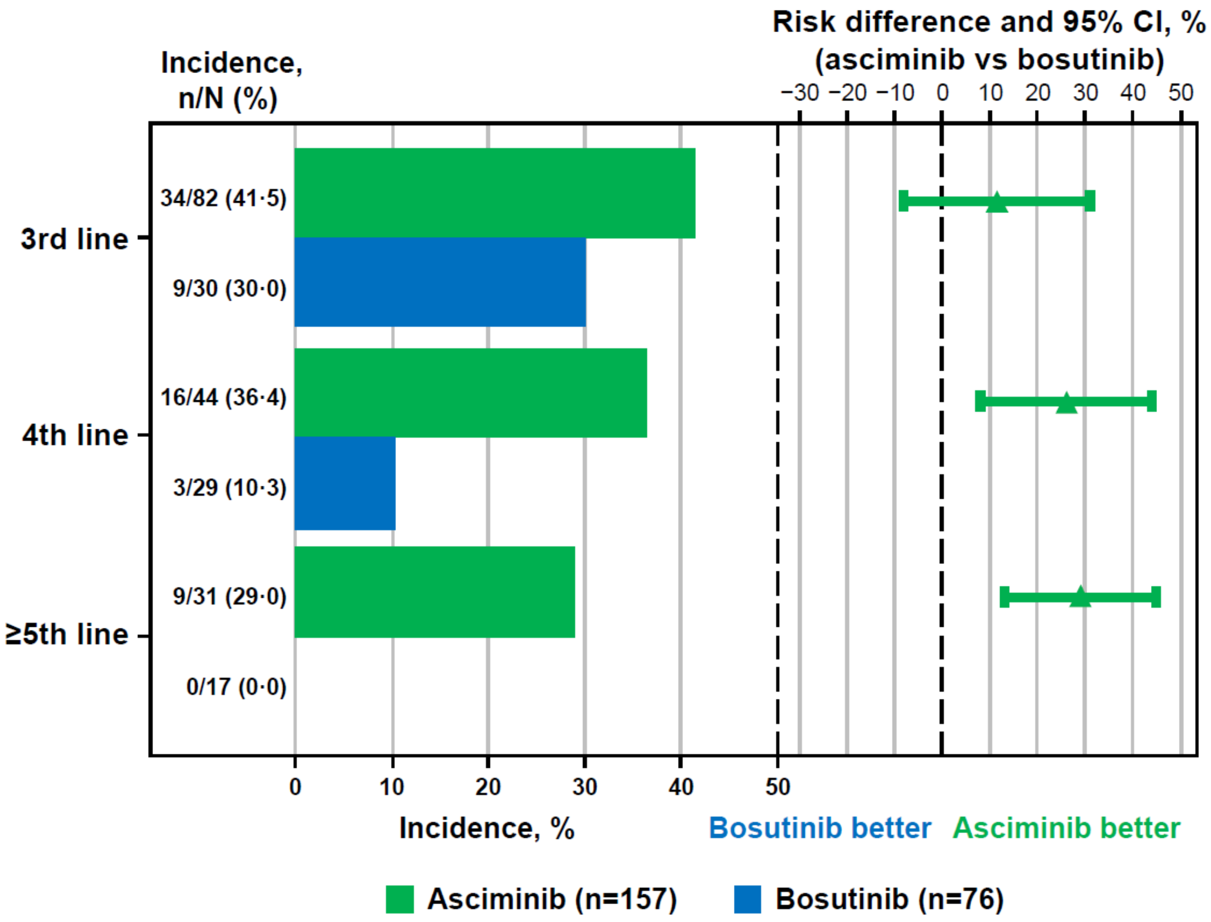

MMR, major molecular response (*BCR::ABL1*^{IS} ≤0.1% on the International Scale).

Figure S3: Time to treatment failure*

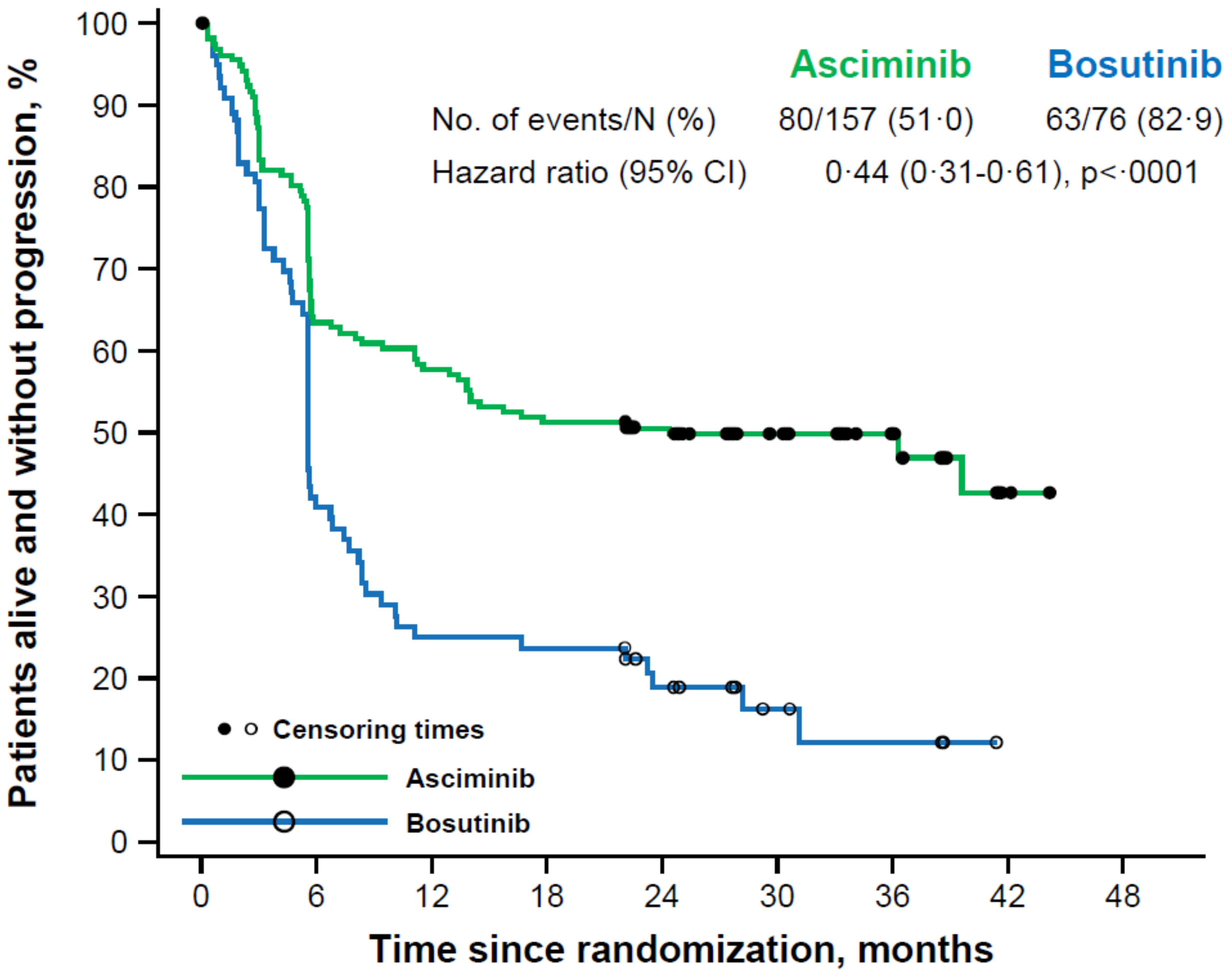

Number of patients still at risk: events

**Asciminib**	157:0	99:57	90:66	80:76	64:77	38:78	18:78	2:80	0:80
**Bosutinib**	76:0	31:45	19:57	18:58	11:61	5:62	3:63	0:63	0:63

* The time from date of randomization to an event of treatment failure (defined as lack of efficacy or discontinuation for any reason).

Figure S4: MDASI-CML MMRM mean change (95% CI) from baseline to week 96: symptom and interference items

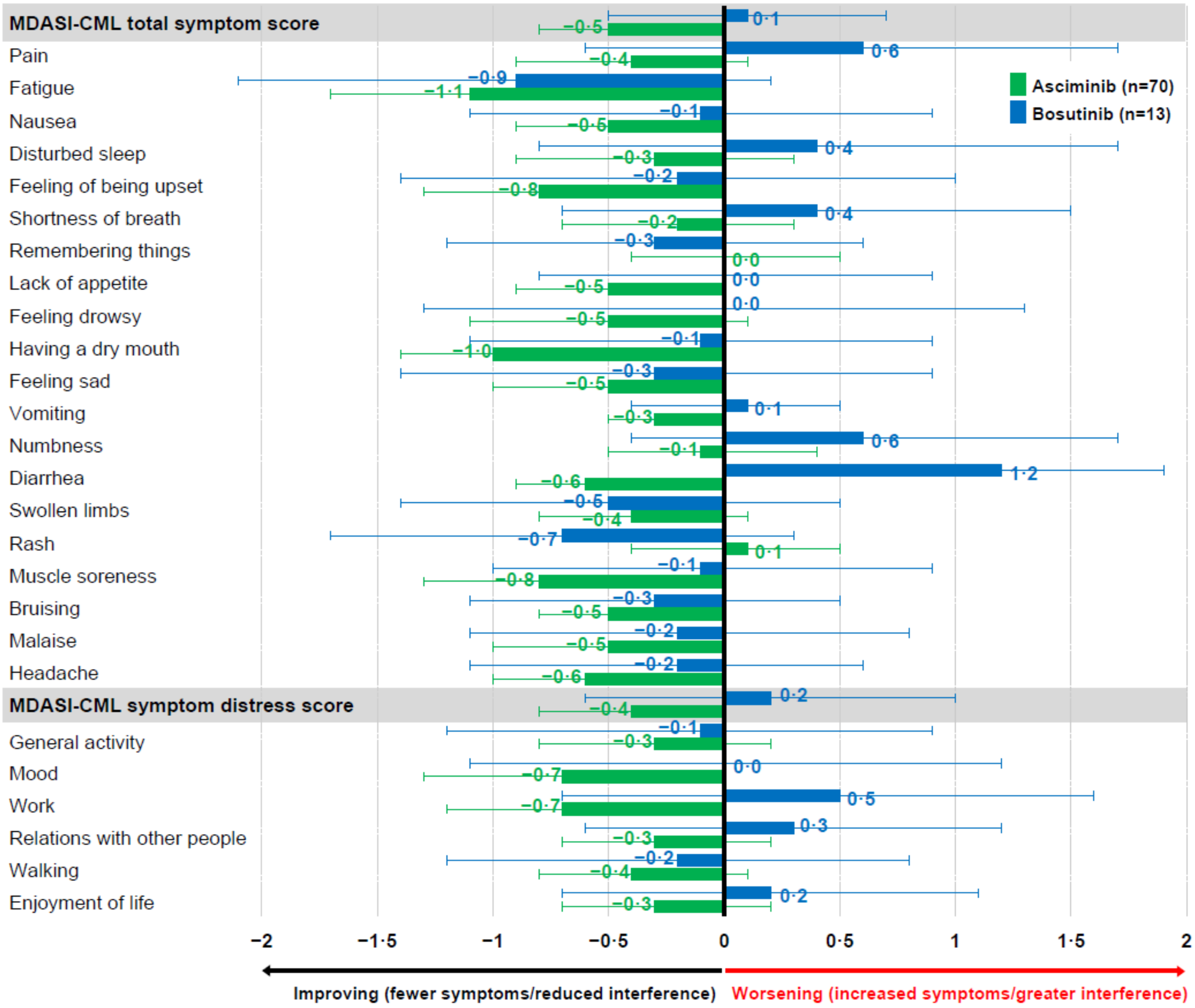

MDASI, MD Anderson Symptom Interference; MMRM, mixed-effect model for repeated measures.

Tables

Table S1: Analysis sets

Patients, n (%)	Asciminib 40 mg twice daily (n=157)	Bosutinib 500 mg once daily (n=76)
Full analysis set[a]	157 (100.0)	76 (100.0)
Safety set[b]	156 (99.4)	76 (100.0)
CCyR analysis set[c]	103 (65.6)	62 (81.6)
BCR::ABL1^{IS} ≤1% analysis set[d]	142 (90.4)	72 (94.7)

CCyR, complete cytogenetic response.
[a] Includes all patients for whom study treatment has been assigned by randomization.
[b] Includes all patients who received at least one dose of study treatment. Patients were analyzed according to the actual study treatment received: randomized treatment if patients took at least one dose of treatment or first treatment received if randomized treatment was never received. There were 156 patients in the asciminib arm in the safety set, because one patient developed cytopenia after randomization and was not treated per investigator's decision.
[c] Includes patients from the full analysis set who are not in CCyR at baseline. Patients in CCyR (asciminib, n=19; bosutinib, n=5) or with non-evaluable or missing bone marrow assessments (asciminib, n=35; bosutinib, n=9) are not part of the CCyR analysis set.
[d] Includes patients from the full analysis set who are not at *BCR::ABL1*^{IS} ≤1% at baseline.

Table S2: Demographic and clinical characteristics of patients at baseline

**Variable**	**Asciminib 40 mg twice daily (n=157)**	**Bosutinib 500 mg once daily (n=76)**	**All patients (N=233)**
Median age (range), y	52.0 (24-83)	52.0 (19-77)	52.0 (19-83)
Female sex, n (%)	75 (47.8)	45 (59.2)	120 (51.5)
Male sex, n (%)	82 (52.2)	31 (40.8)	113 (48.5)
Race, n (%)			
White	118 (75.2)	56 (73.7)	174 (74.7)
Asian	22 (14.0)	11 (14.5)	33 (14.2)
Black or African American	8 (5.1)	2 (2.6)	10 (4.3)
Native American	1 (0.6)	0	1 (0.4)
Other	5 (3.2)	7 (9.2)	12 (5.2)
Unknown	3 (1.9)	0	3 (1.3)
Ethnicity, n (%)			
Hispanic or Latino	15 (9.6)	17 (22.4)	32 (13.7)
Not Hispanic or Latino	102 (65.0)	43 (56.6)	145 (62.2)
Not reported	23 (14.6)	11 (14.5)	34 (14.6)
Unknown	17 (10.8)	5 (6.6)	22 (9.4)
ECOG performance status, n (%)			
0	126 (80.3)	62 (81.6)	188 (80.7)
1	28 (17.8)	14 (18.4)	42 (18.0)
2	2 (1.3)	0	2 (0.9)
Missing	1 (0.6)	0	1 (0.4)
MCyR	46 (29.3)	22 (28.9)	68 (29.2)
Prior TKIs, n (%)			
Imatinib	130 (82.8)	63 (82.9)	193 (82.8)
Nilotinib	104 (66.2)	56 (73.7)	160 (68.7)
Dasatinib	131 (83.4)	65 (85.5)	196 (84.1)
Ponatinib	23 (14.6)	18 (23.7)	41 (17.6)
Radotinib	4 (2.5)	2 (2.6)	6 (2.6)
Other	5 (3.2)	4 (5.3)	9 (3.9)
Number of lines of prior TKI therapy, n (%)[a]			
2	82 (52.2)	30 (39.5)	112 (48.1)
3	44 (28.0)	29 (38.2)	73 (31.3)
4	24 (15.3)	10 (13.2)	34 (14.6)
≥5	7 (4.5)	7 (9.2)	14 (6.0)
Reason for discontinuation of last TKI, n (%)			
Lack of efficacy[b]	95 (60.5)	54 (71.1)	149 (63.9)
Lack of tolerability	59 (37.6)	22 (28.9)	81 (34.8)
Other[c]	3 (1.9)	0	3 (1.3)
BCR::ABL1^{IS} at baseline, n (%)			
>0.1% to ≤1%[d]	15 (9.6)	4 (5.3)	NA
>1% to ≤10%	45 (28.7)	23 (30.3)	NA

>10%	97 (61.8)	49 (64.5)	NA
Patients with any *BCR::ABL1* mutation, n (%)	20 (12.7)	10 (13.2)	30 (12.9)
Patients with multiple *BCR::ABL1* mutations, n (%)	3 (1.9)	0	3 (1.3)

Reprinted from Rea D, et al. *Blood* 2021; **138:** 2031–2041. Copyright © 2021 American Society of Hematology.
ECOG, Eastern Cooperative Oncology Group; NA, not available.
[a] The number of lines of prior TKI therapy was based on the sequence of treatments.
[b] Lack of efficacy criteria were based on 2013 ELN recommendations (see Supplemental Appendix for details).
[c] Includes study medication wrongly assigned, lack of efficacy and tolerability, and optimal response not reached after 5 years of treatment.
[d] All patients with *BCR::ABL1*^{IS} <1% at baseline were intolerant to the last TKI, except 1 in the asciminib arm (who deviated from the protocol).

Table S3: MMR and *BCR::ABL1*[IS] ≤1% at and by time points

	MMR				*BCR::ABL1*[IS] ≤1%			
	At time points[c]		By time points[d]		At time points[c]		By time points[d]	
	Asciminib 40 mg twice daily (n=157)	Bosutinib 500 mg once daily (n=76)	Asciminib 40 mg twice daily (n=157)	Bosutinib 500 mg once daily (n=76)	Asciminib 40 mg twice daily (n=142)	Bosutinib 500 mg once daily (n=72)	Asciminib 40 mg twice daily (n=142)	Bosutinib 500 mg once daily (n=72)
**Week 24**								
Response, n (%)	40 (25.48)	10 (13.16)	43 (27.39)	11 (14.47)	63 (44.37)	15 (20.83)	67 (47.18)	21 (29.17)
95% CI for response[a]	(18.87-33.04)	(6.49-22.87)	(20.58-35.07)	(7.45-24.42)	(36.04-52.93)	(12.16-32.02)	(38.76-55.73)	(19.05-41.07)
Common risk difference, % (95% CI)[b]	12.24 (2.19-22.30)		12.85 (2.40-23.29)		23.92 (11.36-36.49)		18.43 (5.21-31.65)	
**Week 48**								
Response, n (%)	46 (29.30)	10 (13.16)	55 (35.03)	15 (19.74)	61 (42.96)	14 (19.44)	72 (50.70)	24 (33.33)
95% CI for response[a]	(22.32-37.08)	(6.49-22.87)	(27.60-43.04)	(11.49-30.46)	(34.69-51.53)	(11.06-30.47)	(42.19-59.19)	(22.66-45.43)
Common risk difference, % (95% CI)[b]	16.09 (5.69-26.49)		15.24 (3.65-26.83)		23.85 (11.36-36.33)		17.79 (4.29-31.29)	
**Week 96**								
Response, n (%)	59 (37.58)	12 (15.79)	67 (42.68)	18 (23.68)	64 (45.07)	14 (19.44)	75 (52.82)	24 (33.33)
95% CI for response[a]	(29.99-45.65)	(8.43-25.96)	(34.83-50.81)	(14.68-34.82)	(36.72-53.64)	(11.06-30.47)	(44.27-61.24)	(22.66-45.43)
Common risk difference, % (95% CI)[b]	21.74 (10.53-32.95)		18.93 (6.61-31.25)		26.02 (13.48-38.56)		19.92 (6.43-33.41)	

MMR, major molecular response (*BCR::ABL1*[IS] ≤0.1% on the International Scale).
[a] Clopper-Pearson 95% two-sided CI for response rate.
[b] The common risk difference, after adjusting for stratum: baseline major cytogenetic response status (based on randomization data) and its 95% CI, was estimated using the Mantel-Haenszel method.
[c] Response rates at a given time point were calculated based on the number of patients with a response at this time point, regardless of whether they had previously achieved a response.

[d] Response rates by a given time point were calculated based on the cumulative number of patients who achieved a response at any time up to this time point.

Table S4: MR^4 and $MR^{4.5}$ rates at weeks 24 and 96

n (%)	At week 24		At week 96	
	**Asciminib 40 mg twice daily (n=157)**	**Bosutinib 500 mg once daily (n=76)**	**Asciminib 40 mg twice daily (n=157)**	**Bosutinib 500 mg once daily (n=76)**
MR^4	17 (10.8)	4 (5.3)	27 (17.2)	8 (10.5)
$MR^{4.5}$	14 (8.9)	1 (1.3)	17 (10.8)	4 (5.3)

IS, International Scale; MR^4, *BCR::ABL1*^{IS} ≤0.01%; $MR^{4.5}$, *BCR::ABL1*^{IS} ≤0.0032%.

Table S5: *BCR::ABL1* mutations at baseline and best response by data cutoff

**Mutation at baseline**[a]	**Asciminib 40 mg twice daily**				**Bosutinib 500 mg once daily**			
	**All patients, n**	**Best response**	**Discontinued treatment**	**Mutations at end of treatment**	**All patients, n**	**Best response**	**Discontinued treatment**	**Mutations at end of treatment**
**Patients with any mutation**	**17**		**10**	**9**	**8**		**6**	**5**
G250E[b]	1	MMR or better	No	–	–	–	–	–
	1	MMR or better	No	–	–	–	–	–
Y253H[b]	1	*BCR::ABL1*^{IS} >10%	Yes	Y253H	–	–	–	–
	1	MMR or better	No	–	–	–	–	–
E255K[b]	1	MMR or better	No	–	–	–	–	–
	1	MMR or better	No	–	–	–	–	–
E255V[b]	1	MMR or better	No	–	1	*BCR::ABL1*^{IS} >10%	Yes	E255V
F317L[b]	1	*BCR::ABL1*^{IS} >1% to ≤10%	Yes	E355G	1	*BCR::ABL1*^{IS} >1% to ≤10%	Yes	F317L
	1	*BCR::ABL1*^{IS} >10%	Yes	F317L	1	MMR or better	No	–
F359C[b]	1	*BCR::ABL1*^{IS} >10%	Yes	F359C	–	–	–	–
F359V[b]	1	*BCR::ABL1*^{IS} >1% to ≤10%	Yes	F359V	–	–	–	–
	2	*BCR::ABL1*^{IS} >10%	Yes	F359V	–	–	–	–
E459K[c]	1	*BCR::ABL1*^{IS} >10%	Yes	–	–	–	–	–
W478R[d]	1	MMR or better	No	–	–	–	–	–
L248V/F317L[b]	1	*BCR::ABL1*^{IS} >0.1% to ≤1%	Yes	F317L	–	–	–	–
Y253H[b]/F486S[d]	1	*BCR::ABL1*^{IS} >10%	Yes	M244V	–	–	–	–
M244V[b]	–	–	–	–	2	*BCR::ABL1*^{IS} >10%	Yes	M244V
Q252H[b]	–	–	–	–	1	*BCR::ABL1*^{IS} >10%	Yes	Q252H
F359I[b]	–	–	–	–	1	MMR or better	No	–
R473Q[d]	–	–	–	–	1	MMR or better	Yes	–

MMR, major molecular response (*BCR::ABL1*^{IS} ≤0.1% on the International Scale).

[a] Mutations were determined by Sanger sequencing. Patients with T315I and V299L *BCR::ABL1* mutations identified at week 1 day 1 were discontinued from study treatment per protocol.

[b] Adenosine triphosphate–binding region.

[c] Myristoyl pocket region at a residue that is not in direct contact with asciminib.

[d] Kinase C-terminal or core regions.

Table S6: *BCR::ABL1* mutations at the end of treatment in patients who discontinued treatment

**n (%)**[a]	**Asciminib 40 mg twice daily**	**Bosutinib 500 mg once daily**
**Lack of efficacy or disease progression**	**39**	**30**
No mutations detected at end of treatment	22 (56.4)	20 (66.7)
Missing assessments at end of treatment	1 (2.6)	3 (10.0)
Mutations detected at end of treatment	16 (41.0)	7 (23.3)
Newly emerging mutations at end of treatment	10 (25.6)	2 (6.7)
ATP-binding site	M244V (n=3)[b]<br>E355G (n=1)[c]<br>F359V (n=1)<br>T315I (n=1)	T315I (n=1)<br>V299L (n=1)
Myristoyl pocket	A337T (n=3)<br>P465S (n=1)	None
Mutations at baseline and end of treatment	6 (15.4)	5 (16.7)
ATP-binding site	F317L (n=2)<br>F359C/V (n=3)<br>Y253H (n=1)	M244V (n=2)<br>E255V (n=1)<br>F317L (n=1)<br>Q252H (n=1)
**Other**[d]	33	31
Mutations identified at end of treatment	3 (9.1)	2 (6.5)
Newly emerging mutations	0	0
Mutations at baseline and end of treatment	3 (9.1)	2 (6.5)
ATP-binding site	F359V (n=1)<br>T315I (n=2)	T315I (n=1)<br>V299L (n=1)
Myristoyl pocket	E462K (n=1)	None
No mutations identified at end of treatment	16 (48.5)	14 (45.2)
No mutation assessment at end of treatment	14 (42.4)	15 (48.4)

ATP, adenosine triphosphate.

[a] Patients with T315I and V299L *BCR::ABL1* mutations identified at week 1 day 1 were discontinued from study treatment per protocol.

[b] One patient had Y253H and F486S *BCR::ABL1* mutations at baseline that were not detected at the time of discontinuation.

[c] Patient had the F317L *BCR::ABL1* mutation at baseline, which was not detected at the time of discontinuation.

[d] Includes physician decision, adverse events, patient decision, death, lost to follow-up, and protocol deviation.

Table S7: Laboratory abnormalities

Event, n (%)	Asciminib 40 mg twice daily (n=156)		Bosutinib 500 mg once daily (n=76)	
	**All grades**	**Grade 3/4**	**All grades**	**Grade 3/4**
**Hematology abnormalities**				
Decreased hemoglobin	56 (36.6)	3 (2.0)	41 (53.9)	4 (5.3)
Decreased leukocytes	69 (45.1)	14 (9.2)	22 (28.9)	4 (5.3)
Decreased lymphocytes	31 (20.4)	5 (3.3)	26 (34.2)	2 (2.6)
Decreased neutrophils	65 (42.8)	33 (21.7)	25 (32.9)	11 (14.5)
Decreased platelets	71 (46.4)	36 (23.5)	27 (36.0)	9 (12.0)
Increased prothrombin INR	14 (9.2)	1 (0.7)	8 (11.3)	1 (1.4)
**Selected biochemical abnormalities**				
ALT (serum)	40 (25.6)	1 (0.6)	38 (50.0)	12 (15.8)
ALP (serum)	20 (12.8)	0	9 (11.8)	0
Amylase (serum)	20 (12.8)	2 (1.3)	10 (13.2)	0
AST (serum)	33 (21.2)	3 (1.9)	35 (46.1)	5 (6.6)
Bilirubin (serum)	18 (11.5)	0	3 (3.9)	0
Cholesterol (serum)	19 (12.2)	0	6 (7.9)	0
Creatine kinase	46 (29.5)	4 (2.6)	18 (23.7)	4 (5.3)
Creatinine (plasma/serum)	24 (15.4)	0	20 (26.3)	0
Glucose (serum)	89 (57.1)	6 (3.8)	36 (47.4)	2 (2.6)
Pancreatic lipase	24 (15.4)	7 (4.5)	14 (18.4)	5 (6.6)
Triglycerides (plasma/serum)	69 (44.2)	8 (5.1)	23 (30.3)	2 (2.6)
Urate (serum)	32 (20.5)	9 (5.8)	14 (18.4)	2 (2.6)

INR, international normalized ratio.

Table S8: Adverse events leading to treatment discontinuation of study treatment by preferred term

Event, n (%)[a]	Asciminib 40 mg twice daily (n=156)		Bosutinib 500 mg once daily (n=76)	
	All grades	Grade ≥3	All grades	Grade ≥3
Number of patients with ≥1 adverse event	12 (7.7)	12 (7.7)	20 (26.3)	15 (19.7)
Thrombocytopenia[b]	5 (3.2)	5 (3.2)	1 (1.3)	1 (1.3)
Neutropenia[c]	4 (2.6)	4 (2.6)	3 (3.9)	3 (3.9)
Lipase increased	3 (1.9)	3 (1.9)	0	0
Amylase increased	1 (0.6)	1 (0.6)	0	0
Cerebral disorder[d]	1 (0.6)	1 (0.6)	0	0
Ejection fraction decreased	1 (0.6)	1 (0.6)	0	0
Ischemic stroke	1 (0.6)	1 (0.6)	0	0
Alanine aminotransferase increased	0	0	4 (5.3)	3 (3.9)
Aspartate aminotransferase increased	0	0	2 (2.6)	1 (1.3)
Blood creatinine increased	0	0	1 (1.3)	0
Diarrhea	0	0	2 (2.6)	1 (1.3)
Diffuse large B-cell lymphoma	0	0	1 (1.3)	1 (1.3)
Drug eruption	0	0	1 (1.3)	0
Hydrothorax	0	0	1 (1.3)	1 (1.3)
Pleural effusion	0	0	3 (3.9)	2 (2.6)
Pyrexia	0	0	1 (1.3)	1 (1.3)
Rash	0	0	1 (1.3)	1 (1.3)
Squamous cell carcinoma	0	0	1 (1.3)	1 (1.3)

[a] Based on the safety analysis set. Numbers represent counts of patients. A patient with multiple severity grades for an adverse event is only counted under the maximum grade; Medical Dictionary for Regulatory Activities version 24.1, Common Terminology Criteria for Adverse Events version 4.03.
[b] Includes thrombocytopenia and decreased platelet count.
[c] Includes neutropenia and decreased neutrophil count.
[d] Patient was hospitalized as a result of cerebral infarction, with cerebral disorder reported as the final cause of permanent discontinuation.

Table S9: Dose adjustments and discontinuation of study drug

Category[a,b]	Asciminib 40 mg twice daily (n=156)	Bosutinib 500 mg once daily[c] (n=76)
**Patients with dose reduction, n (%)**		
No dose reduction	91 (58.3)	41 (53.9)
≥1 dose reduction	65 (41.7)	35 (46.1)
Only 1 dose reduction	40 (25.6)	17 (22.4)
2 dose reductions	14 (9.0)	16 (21.1)
>2 dose reductions	11 (7.1)	2 (2.6)
Patients with ≥1 dose reduction by reason, n (%)		
Adverse event	37 (23.7)	34 (44.7)
Dosing error	31 (19.9)	1 (1.3)
Physician decision	2 (1.3)	2 (2.6)
Technical problems	1 (0.6)	0
Dispensing error	0	1 (1.3)
Median dose reduction per patient (range), n	1.0 (1-67)	2.0 (1-3)
**Patients with dose interruption, n (%)**		
No dose interruption	69 (44.2)	20 (26.3)
≥1 dose interruption	87 (55.8)	56 (73.7)
Only 1 dose interruption	40 (25.6)	26 (34.2)
2 Dose interruptions	19 (12.2)	14 (18.4)
>2 Dose interruptions	28 (17.9)	16 (21.1)
Patients with ≥1 dose interruption by reason, n (%)		
Adverse event	66 (42.3)	47 (61.8)
Dosing error	21 (13.5)	13 (17.1)
Physician decision	8 (5.1)	5 (6.6)
Patient decision	8 (5.1)	3 (3.9)
Dispensing error	2 (1.3)	1 (1.3)
Technical problems	1 (0.6)	0
Median dose interruption per patient (range), n	2.0 (1-25)	2.0 (1-11)
Median duration of dose interruption (range), days	27.0 (1-260)	21.0 (1-128)
**Patients with dose increase, n (%)**		
No dose increase	154 (98.7)	70 (92.1)
≥1 dose increase	2 (1.3)	6 (7.9)
Only 1 dose increase	0	6 (7.9)
>2 dose increases	2 (1.3)	0
Patients with ≥1 dose increase by reason, n (%)		
As per protocol	0	5 (6.6)
Dosing error	2 (1.3)	1 (1.3)
Median dose increase per patient (range), n	5.5 (4-7)	1.0 (1-1)
Permanent discontinuation, n (%)	72 (46.2)	62 (81.6)

[a] Based on the safety analysis set.
[b] Per protocol, adverse events were managed first by dose interruption and upon resolution, dose level was maintained or reduced, or study treatment was permanently discontinued, depending on the type of event and its duration.
[c] Per protocol, patients randomized to bosutinib still on 500 mg once daily could have their dose escalated to 600 mg once daily if complete hematologic recovery had not been reached by week 8 or complete cytogenetic response by week 12 in patients without grade ≥3 adverse events.

Table S10: Exposure-adjusted incidence rate of non-hematologic adverse events by preferred term (reported in ≥5% of patients in any treatment arm as shown in Table 2)

Exposure-adjusted incidence, n (IR per 100 patient-treatment years)[a]	Asciminib 40 mg twice daily (n=156)		Bosutinib 500 mg once daily (n=76)	
	All grades	Grade ≥3	All grades	Grade ≥3
**Patients with ≥1 event**	**142 (371.6)**	**88 (55.8)**	**74 (1440.8)**	**52 (115.8)**
Headache	31 (14.3)	3 (1.1)	12 (19.2)	0
Fatigue	23 (9.8)	1 (0.4)	7 (10.2)	1 (1.3)
Hypertension	21 (8.3)	10 (3.8)	4 (5.3)	3 (4.0)
Arthralgia	20 (8.4)	1 (0.4)	3 (4.1)	0
Diarrhea	20 (8.0)	0	55 (234.4)	8 (11.2)
Nausea	18 (7.4)	1 (0.4)	35 (83.3)	0
Nasopharyngitis	17 (6.9)	0	3 (4.0)	0
Abdominal pain	14 (5.4)	0	12 (18.5)	1 (1.3)
Pain in extremity	14 (5.5)	1 (0.4)	5 (6.8)	0
Rash	14 (5.5)	0	18 (31.9)	3 (4.0)
Asthenia	13 (5.1)	0	1 (1.4)	0
Cough	13 (5.0)	0	5 (6.9)	0
Vomiting	12 (4.7)	2 (0.7)	20 (32.5)	0
Upper respiratory tract infection	11 (4.2)	1 (0.4)	4 (5.6)	0
Amylase increased	9 (3.4)	1 (0.4)	4 (5.8)	0
Aspartate aminotransferase increased	9 (3.5)	3 (1.1)	16 (23.3)	5 (6.7)
Constipation	8 (3.0)	0	4 (5.3)	0
Decreased appetite	8 (3.1)	0	6 (8.0)	0
Dry skin	8 (3.0)	0	6 (8.3)	0
Dyspnea	8 (3.0)	0	4 (5.4)	0
Lipase increased	8 (3.0)	6 (2.3)	5 (6.8)	4 (5.4)
Pruritus	8 (3.1)	0	5 (6.8)	1 (1.3)
Abdominal pain upper	7 (2.7)	0	5 (6.8)	1 (1.3)
Alanine aminotransferase increased	7 (2.7)	1 (0.4)	23 (36.7)	11 (15.6)
Pyrexia	6 (2.2)	2 (0.7)	6 (8.2)	1 (1.3)

Blood creatinine increased	5 (1.9)	0	5 (6.9)	0
Influenza like illness	3 (1.1)	0	4 (5.6)	0
Hypophosphatemia	2 (0.7)	1 (0.4)	4 (5.4)	3 (4.0)

IR, incidence rate.

[a] Based on the safety analysis set. The exposure-adjusted IR was calculated by dividing the number of patients with an event by the corresponding sum of the exposure duration for all patients, where duration of exposure in 100 patient-treatment years was counted up to the first qualifying event (or end of time at risk for patients without the event).

Table S11: Arterial-occlusive events

Category	Asciminib 40 mg twice daily (n=156)	Bosutinib 500 mg once daily (n=76)
**Patients with AOEs, n (%)**	8 (5.1)	1 (1.3)
**Patients with events observed by the week 24 cutoff, n (%)**		
Myocardial ischemia	2 (1.3)	0
Acute coronary syndrome	0	1 (1.3)
Coronary artery disease	1 (0.6)	0
Ischemic stroke	1 (0.6)	0
Mesenteric artery embolism/thrombosis	1 (0.6)	0
**Additional patients with events since the week 24 cutoff, n (%)**		
Cerebral infarction	1 (0.6)	0
Myocardial infarction	1 (0.6)	0
Troponin increased	1 (0.6)	0
**Exposure-adjusted AOE incidence rate (per 100 patient treatment-years)**		
Primary analysis (week 24)	3.3	2.0
Current analysis	3.0	1.4

AOE, arterial-occlusive event.

Table S12: Characteristics of patients with arterial-occlusive events

Arm	Patient	Age, years	Sex	AE term	Study day of occurrence	CV risk factors at screening	Last prior TKIs
**Asciminib**	1	71	F	Myocardial ischemia	1	No relevant medical history	Imatinib and nilotinib
	2	52	F	Myocardial ischemia	26	Former smoker, hypertension, low levels of physical activity, unhealthy diet	Nilotinib, dasatinib, imatinib, and ponatinib
	3	65	M	Ischemic stroke	56	Hypertension and left ventricular hypertrophy per ECG	Imatinib, nilotinib, and dasatinib
	4	59	M	Coronary artery disease	197	Hypertension and hyperlipidemia. Chronic cardiac failure reported as part of medical history	Imatinib and nilotinib
	5	57	M	Myocardial infarction	253	No relevant medical history	Imatinib, dasatinib, and nilotinib
	6	62	F	Mesenteric artery embolism/thrombosis	260	Hypertension and low levels of physical activity	Imatinib, dasatinib, and nilotinib. On study day 245 the patient discontinued asciminib and on day 253 started ponatinib
	7	70	M	Cerebral infarction	276	Hypertension and hyperlipidemia	Nilotinib, dasatinib, and ponatinib
	8	63	M	Troponin increased	612	Prior medical conditions included implantable defibrillation insertion. Active conditions included myotonic dystrophy, coronary artery disease, ejection fraction decreased, and hyperlipidemia	Dasatinib and imatinib
**Bosutinib**	1	67	M	Acute coronary syndrome	173	Prior myocardial infarction, hypertension, and hyperlipidemia	Imatinib, ponatinib, and nilotinib

AE, adverse event; CV, cardiovascular; ECG, electrocardiogram; TKI, tyrosine kinase inhibitor.
