# Supplementary material for: Asciminib vs bosutinib in chronic-phase chronic myeloid leukemia previously treated with at least two tyrosine kinase inhibitors: longer-term follow-up of ASCEMBL
Source: Leukemia. 2023 Jan 30;37(3):617–26. doi: 10.1038/s41375-023-01829-9 (PMC9991909; doi:10.1038/s41375-023-01829-9)
Supplement: Supplementary file 2 — Figure S1 [file 41375_2023_1829_MOESM2_ESM.pdf]

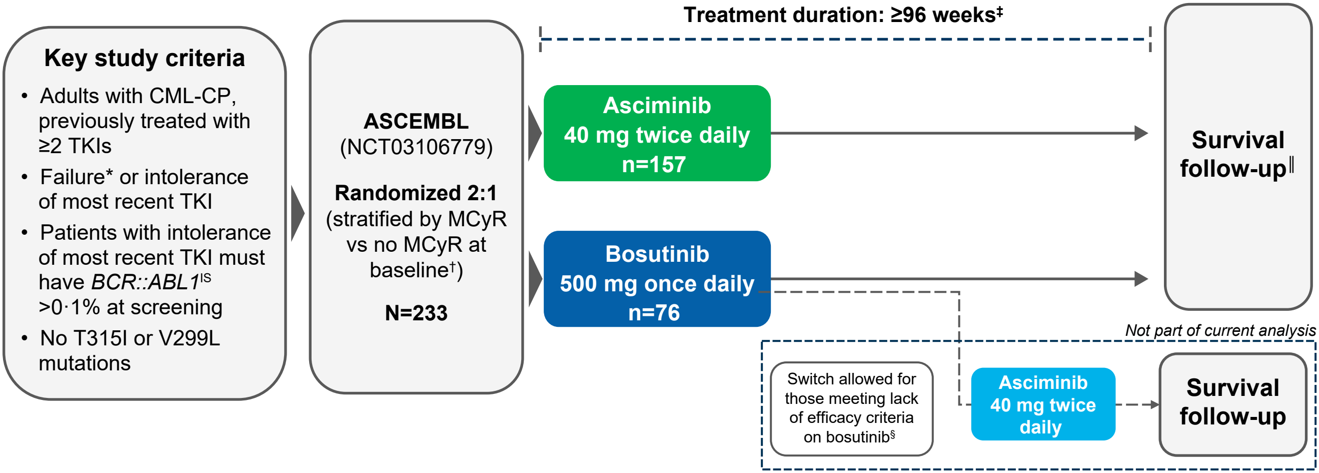
Treatment duration: ≥96 weeks‡
Key study criteria
• Adults with CML-CP, previously treated with ≥2 TKIs
• Failure* or intolerance of most recent TKI
• Patients with intolerance of most recent TKI must have BCR::ABL1IS >0·1% at screening
• No T315I or V299L mutations
ASCEMBL
(NCT03106779)
Randomized 2:1
(stratified by MCyR vs no MCyR at baseline†)
N=233
Asciminib
40 mg twice daily
n=157
Bosutinib
500 mg once daily
n=76
Survival follow-up‖
Not part of current analysis
Switch allowed for those meeting lack of efficacy criteria on bosutinib§
Asciminib
40 mg twice daily
Survival follow-up
