# Supplementary figures and images for: Asciminib vs bosutinib in chronic-phase chronic myeloid leukemia previously treated with at least two tyrosine kinase inhibitors: longer-term follow-up of ASCEMBL
Source: Leukemia. 2023 Jan 30;37(3):617–26. doi: 10.1038/s41375-023-01829-9 (PMC9991909; doi:10.1038/s41375-023-01829-9)

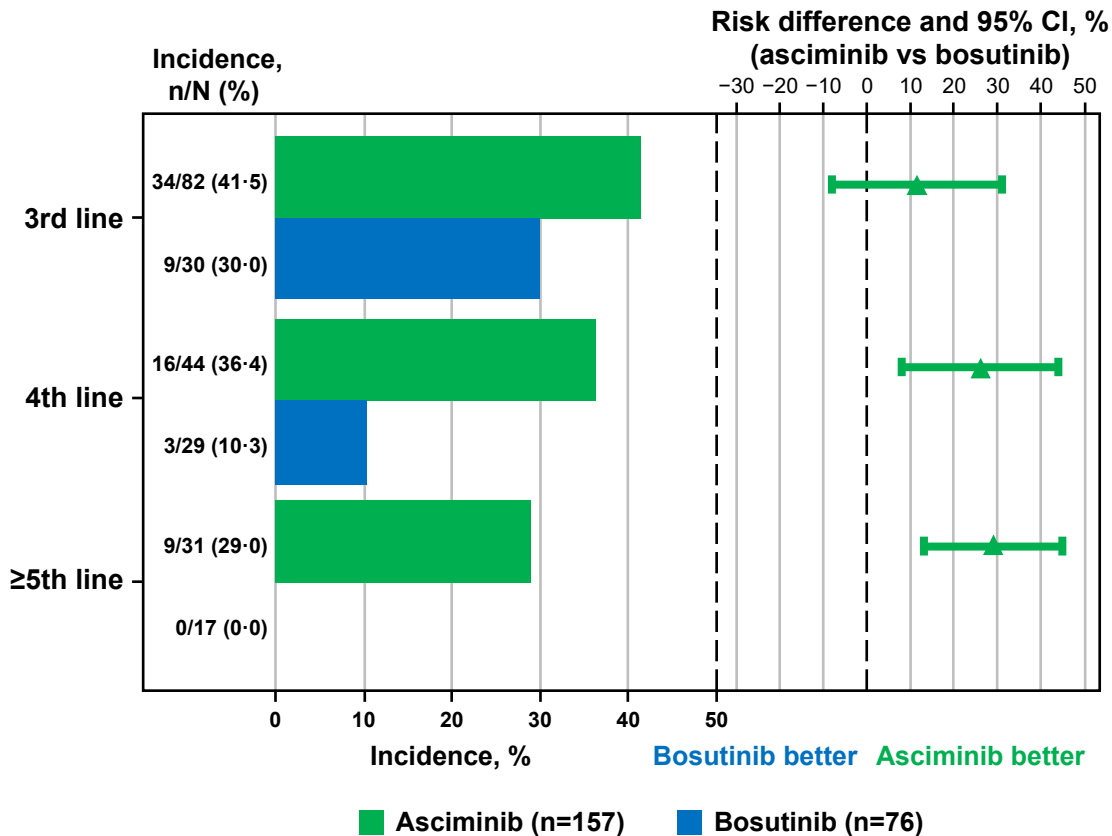

Supplement: Supplementary file 3 — Figure S2 [file 41375_2023_1829_MOESM3_ESM.pdf]

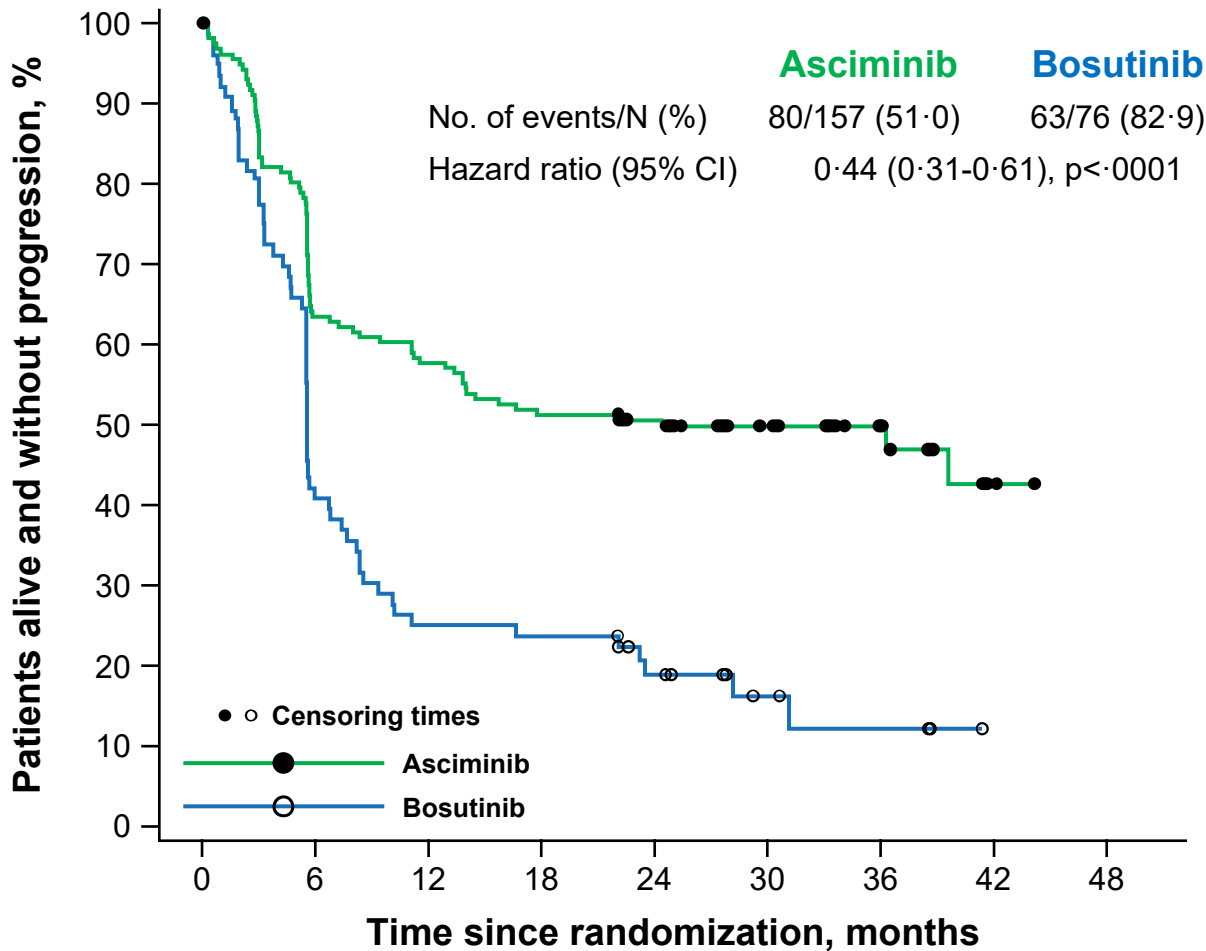

**Number of patients still at risk: events**

|                  |       |       |       |       |       |       |       |      |      |
|------------------|-------|-------|-------|-------|-------|-------|-------|------|------|
| <b>Asciminib</b> | 157:0 | 99:57 | 90:66 | 80:76 | 64:77 | 38:78 | 18:78 | 2:80 | 0:80 |
| <b>Bosutinib</b> | 76:0  | 31:45 | 19:57 | 18:58 | 11:61 | 5:62  | 3:63  | 0:63 | 0:63 |

Supplement: Supplementary file 4 — Figure S3 [file 41375_2023_1829_MOESM4_ESM.pdf]

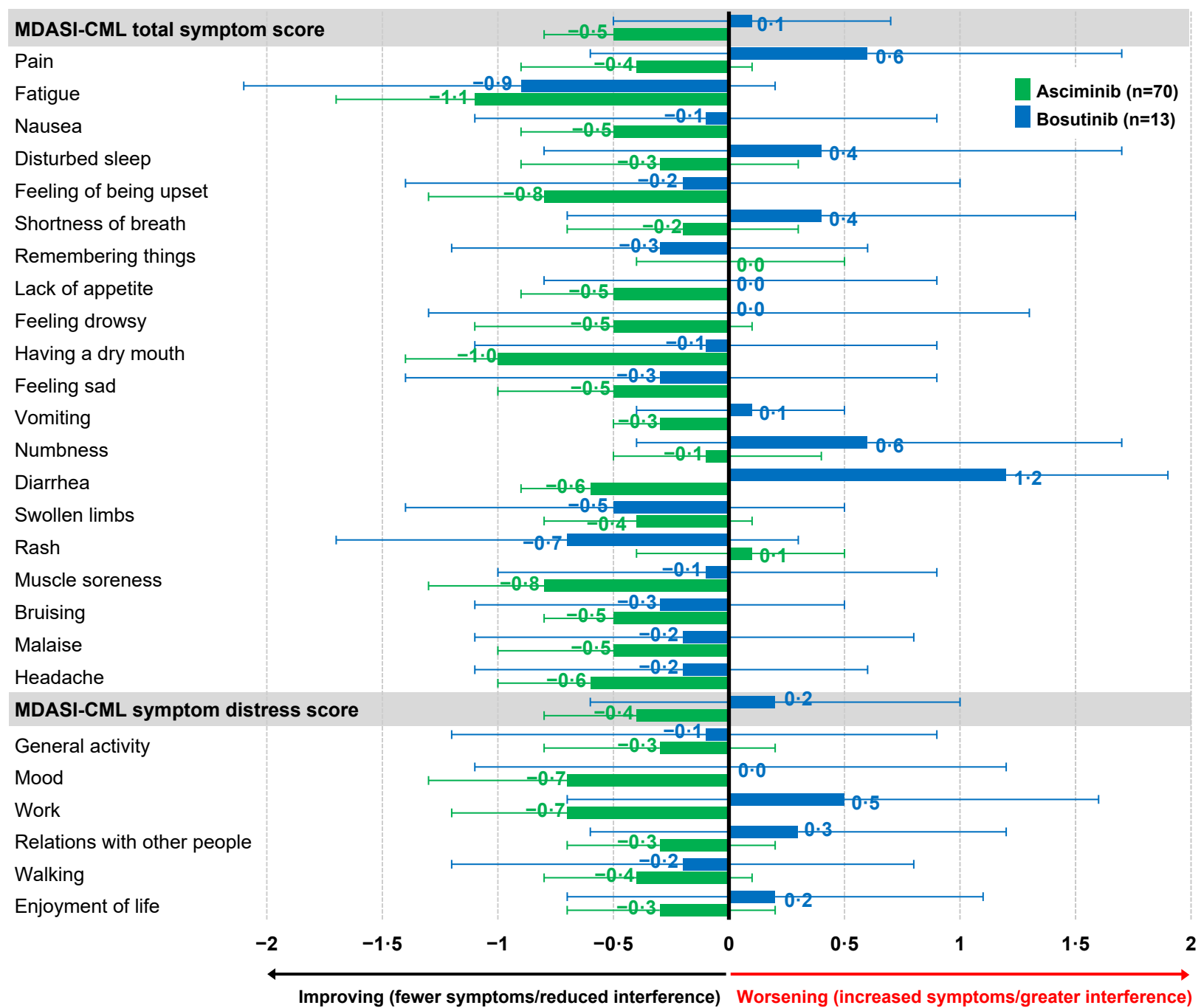

Supplement: Supplementary file 5 — Figure S4 [file 41375_2023_1829_MOESM5_ESM.pdf]
