# Supplementary material for: Asciminib vs bosutinib in chronic-phase chronic myeloid leukemia previously treated with at least two tyrosine kinase inhibitors: longer-term follow-up of ASCEMBL
Source: Leukemia. 2023 Jan 30;37(3):617–26. doi: 10.1038/s41375-023-01829-9 (PMC9991909; doi:10.1038/s41375-023-01829-9)
Supplement: Supplementary file 6 — Table S1 [file 41375_2023_1829_MOESM6_ESM.docx]

**Table S1: Analysis sets**

| **Patients, n (%)** | **Asciminib 40 mg twice daily**  **(n=157)** | **Bosutinib 500 mg once daily**  **(n=76)** |
| --- | --- | --- |
| Full analysis set^a^ | 157 (100.0) | 76 (100.0) |
| Safety set^b^ | 156 (99.4) | 76 (100.0) |
| CCyR analysis set^c^ | 103 (65.6) | 62 (81.6) |
| *BCR::ABL1*^IS^ ≤1% analysis set^d^ | 142 (90.4) | 72 (94.7) |

CCyR, complete cytogenetic response. ^a^ Includes all patients for whom study treatment has been assigned by randomization.

^b^ Includes all patients who received at least one dose of study treatment. Patients were analyzed according to the actual study treatment received: randomized treatment if patients took at least one dose of treatment or first treatment received if randomized treatment was never received. There were 156 patients in the asciminib arm in the safety set, because one patient developed cytopenia after randomization and was not treated per investigator’s decision.

^c^ Includes patients from the full analysis set who are not in CCyR at baseline. Patients in CCyR (asciminib, n=19; bosutinib, n=5) or with non-evaluable or missing bone marrow assessments (asciminib, n=35; bosutinib, n=9) are not part of the CCyR analysis set.

^d^ Includes patients from the full analysis set who are not at *BCR::ABL1*^IS^ ≤1% at baseline.
