# Supplementary material for: Asciminib vs bosutinib in chronic-phase chronic myeloid leukemia previously treated with at least two tyrosine kinase inhibitors: longer-term follow-up of ASCEMBL
Source: Leukemia. 2023 Jan 30;37(3):617–26. doi: 10.1038/s41375-023-01829-9 (PMC9991909; doi:10.1038/s41375-023-01829-9)
Supplement: Supplementary file 7 — Table S2 [file 41375_2023_1829_MOESM7_ESM.docx]

**Table S2: Demographic and clinical characteristics of patients at baseline**

| **Variable** | **Asciminib 40 mg twice daily**  **(n=157)** | **Bosutinib 500 mg once daily**  **(n=76)** | **All patients**  **(N=233)** |
| --- | --- | --- | --- |
| Median age (range), y | 52.0 (24-83) | 52.0 (19-77) | 52.0 (19-83) |
| Female sex, n (%) | 75 (47.8) | 45 (59.2) | 120 (51.5) |
| Male sex, n (%) | 82 (52.2) | 31 (40.8) | 113 (48.5) |
| Race, n (%) |  |  |  |
| White | 118 (75.2) | 56 (73.7) | 174 (74.7) |
| Asian | 22 (14.0) | 11 (14.5) | 33 (14.2) |
| Black or African American | 8 (5.1) | 2 (2.6) | 10 (4.3) |
| Native American | 1 (0.6) | 0 | 1 (0.4) |
| Other | 5 (3.2) | 7 (9.2) | 12 (5.2) |
| Unknown | 3 (1.9) | 0 | 3 (1.3) |
| Ethnicity, n (%) |  |  |  |
| Hispanic or Latino | 15 (9.6) | 17 (22.4) | 32 (13.7) |
| Not Hispanic or Latino | 102 (65.0) | 43 (56.6) | 145 (62.2) |
| Not reported | 23 (14.6) | 11 (14.5) | 34 (14.6) |
| Unknown | 17 (10.8) | 5 (6.6) | 22 (9.4) |
| ECOG performance status, n (%) |  |  |  |
| 0 | 126 (80.3) | 62 (81.6) | 188 (80.7) |
| 1 | 28 (17.8) | 14 (18.4) | 42 (18.0) |
| 2 | 2 (1.3) | 0 | 2 (0.9) |
| Missing | 1 (0.6) | 0 | 1 (0.4) |
| MCyR | 46 (29.3) | 22 (28.9) | 68 (29.2) |
| Prior TKIs, n (%) |  |  |  |
| Imatinib | 130 (82.8) | 63 (82.9) | 193 (82.8) |
| Nilotinib | 104 (66.2) | 56 (73.7) | 160 (68.7) |
| Dasatinib | 131 (83.4) | 65 (85.5) | 196 (84.1) |
| Ponatinib | 23 (14.6) | 18 (23.7) | 41 (17.6) |
| Radotinib | 4 (2.5) | 2 (2.6) | 6 (2.6) |
| Other | 5 (3.2) | 4 (5.3) | 9 (3.9) |
| Number of lines of prior TKI therapy, n (%)^a^ |  |  |  |
| 2 | 82 (52.2) | 30 (39.5) | 112 (48.1) |
| 3 | 44 (28.0) | 29 (38.2) | 73 (31.3) |
| 4 | 24 (15.3) | 10 (13.2) | 34 (14.6) |
| ≥5 | 7 (4.5) | 7 (9.2) | 14 (6.0) |
| Reason for discontinuation of last TKI, n (%) |  |  |  |
| Lack of efficacy^b^ | 95 (60.5) | 54 (71.1) | 149 (63.9) |
| Lack of tolerability | 59 (37.6) | 22 (28.9) | 81 (34.8) |
| Other^c^ | 3 (1.9) | 0 | 3 (1.3) |
| *BCR::ABL1*^IS^ at baseline, n (%) |  |  |  |
| >0.1% to ≤1%^d^ | 15 (9.6) | 4 (5.3) | NA |
| >1% to ≤10% | 45 (28.7) | 23 (30.3) | NA |
| >10% | 97 (61.8) | 49 (64.5) | NA |
| Patients with any *BCR::ABL1* mutation, n (%) | 20 (12.7) | 10 (13.2) | 30 (12.9) |
| Patients with multiple *BCR::ABL1* mutations, n (%) | 3 (1.9) | 0 | 3 (1.3) |

Reprinted from Rea D, et al. *Blood* 2021; **138:** 2031–2041. Copyright © 2021 American Society of Hematology.

ECOG, Eastern Cooperative Oncology Group; NA, not available.

^a^ The number of lines of prior TKI therapy was based on the sequence of treatments.

^b^ Lack of efficacy criteria were based on 2013 ELN recommendations (see Supplemental Appendix for details).

^c^ Includes study medication wrongly assigned, lack of efficacy and tolerability, and optimal response not reached after 5 years of treatment.

^d^ All patients with *BCR::ABL1*^IS^ <1% at baseline were intolerant to the last TKI, except 1 in the asciminib arm (who deviated from the protocol
