# Supplementary material for: Asciminib vs bosutinib in chronic-phase chronic myeloid leukemia previously treated with at least two tyrosine kinase inhibitors: longer-term follow-up of ASCEMBL
Source: Leukemia. 2023 Jan 30;37(3):617–26. doi: 10.1038/s41375-023-01829-9 (PMC9991909; doi:10.1038/s41375-023-01829-9)
Supplement: Supplementary file 8 — Table S3 [file 41375_2023_1829_MOESM8_ESM.docx]

**Table S3: MMR and *BCR::ABL1*^IS^ ≤1% at and by time points**

|  | **MMR** | | | | ***BCR::ABL1*^IS^ ≤1%** | | | |
| --- | --- | --- | --- | --- | --- | --- | --- | --- |
|  | **At time points^c^** | | **By time points^d^** | | **At time points^c^** | | **By time points^d^** | |
|  | **Asciminib 40 mg twice daily**  **(n=157**) | **Bosutinib 500 mg once daily**  **(n=76)** | **Asciminib 40 mg twice daily**  **(n=157)** | **Bosutinib 500 mg once daily**  **(n=76)** | **Asciminib 40 mg twice daily**  **(n=142)** | **Bosutinib 500 mg once daily**  **(n=72)** | **Asciminib 40 mg twice daily**  **(n=142)** | **Bosutinib 500 mg once daily**  **(n=72)** |
| **Week 24** | | | | | | | | |
| Response, n (%) | 40 (25.48) | 10 (13.16) | 43 (27.39) | 11 (14.47) | 63 (44.37) | 15 (20.83) | 67 (47.18) | 21 (29.17) |
| 95% CI for response^a^ | (18.87-33.04) | (6.49-22.87) | (20.58-35.07) | (7.45-24.42) | (36.04-52.93) | (12.16-32.02) | (38.76-55.73) | (19.05-41.07) |
| Common risk difference, % (95% CI)^b^ | 12.24 (2.19-22.30) | | 12.85 (2.40-23.29) | | 23.92 (11.36-36.49) | | 18.43 (5.21-31.65) | |
| **Week 48** | | | | | | | | |
| Response, n (%) | 46 (29.30) | 10 (13.16) | 55 (35.03) | 15 (19.74) | 61 (42.96) | 14 (19.44) | 72 (50.70) | 24 (33.33) |
| 95% CI for response^a^ | (22.32-37.08) | (6.49-22.87) | (27.60-43.04) | (11.49-30.46) | (34.69-51.53) | (11.06-30.47) | (42.19-59.19) | (22.66-45.43) |
| Common risk difference, % (95% CI)^b^ | 16.09 (5.69-26.49) | | 15.24 (3.65-26.83) | | 23.85 (11.36-36.33) | | 17.79 (4.29-31.29) | |
| **Week 96** | | | | | | | | |
| Response, n (%) | 59 (37.58) | 12 (15.79) | 67 (42.68) | 18 (23.68) | 64 (45.07) | 14 (19.44) | 75 (52.82) | 24 (33.33) |
| 95% CI for response^a^ | (29.99-45.65) | (8.43-25.96) | (34.83-50.81) | (14.68-34.82) | (36.72-53.64) | (11.06-30.47) | (44.27-61.24) | (22.66-45.43) |
| Common risk difference, % (95% CI)^b^ | 21.74 (10.53-32.95) | | 18.93 (6.61-31.25) | | 26.02 (13.48-38.56) | | 19.92 (6.43-33.41) | |

MMR, major molecular response (*BCR::ABL1*^IS^ ≤0.1% on the International Scale).
^a^ Clopper-Pearson 95% two-sided CI for response rate.

^b^ The common risk difference, after adjusting for stratum: baseline major cytogenetic response status (based on randomization data) and its 95% CI, was estimated using the Mantel-Haenszel method.

^c^ Response rates at a given time point were calculated based on the number of patients with a response at this time point, regardless of whether they had previously achieved a response.

^d^ Response rates by a given time point were calculated based on the cumulative number of patients who achieved a response at any time up to this time point.
