# Supplementary material for: Asciminib vs bosutinib in chronic-phase chronic myeloid leukemia previously treated with at least two tyrosine kinase inhibitors: longer-term follow-up of ASCEMBL
Source: Leukemia. 2023 Jan 30;37(3):617–26. doi: 10.1038/s41375-023-01829-9 (PMC9991909; doi:10.1038/s41375-023-01829-9)
Supplement: Supplementary file 9 — Table S4 [file 41375_2023_1829_MOESM9_ESM.docx]

**Table S4: MR^4^ and MR^4.5^ rates at weeks 24 and 96**

| **n (%)** | **At week 24** | | **At week 96** | |
| --- | --- | --- | --- | --- |
|  | **Asciminib 40 mg twice daily**  **(n=157)** | **Bosutinib 500 mg once daily**  **(n=76)** | **Asciminib 40 mg twice daily**  **(n=157)** | **Bosutinib 500 mg once daily**  **(n=76)** |
| MR^4^ | 17 (10.8) | 4 (5.3) | 27 (17.2) | 8 (10.5) |
| MR^4.5^ | 14 (8.9) | 1 (1.3) | 17 (10.8) | 4 (5.3) |

IS, International Scale; MR^4^, *BCR::ABL1*^IS^ ≤0.01%; MR^4.5^, *BCR::ABL1*^IS^ ≤0.0032%.
