# Supplementary material for: Asciminib vs bosutinib in chronic-phase chronic myeloid leukemia previously treated with at least two tyrosine kinase inhibitors: longer-term follow-up of ASCEMBL
Source: Leukemia. 2023 Jan 30;37(3):617–26. doi: 10.1038/s41375-023-01829-9 (PMC9991909; doi:10.1038/s41375-023-01829-9)
Supplement: Supplementary file 10 — Table S5 [file 41375_2023_1829_MOESM10_ESM.docx]

**Table S5:** ***BCR::ABL1* mutations at baseline and best response by data cutoff**

| **Mutation at baseline^a^** | **Asciminib 40 mg twice daily** | | | | **Bosutinib 500 mg once daily** | | | | |
| --- | --- | --- | --- | --- | --- | --- | --- | --- | --- |
|  | **All patients, n** | **Best response** | **Discontinued treatment** | **Mutations at end of treatment** | **All patients, n** | **Best response** | **Discontinued treatment** | **Mutations at end of treatment** |  |
| **Patients with any mutation** | **17** |  | **10** | **9** | **8** |  | **6** | **5** |  |
| G250E^b^ | 1 | MMR or better | No | – | – | – | – | – |  |
|  | 1 | MMR or better | No | – | – | – | – | – |  |
| Y253H^b^ | 1 | *BCR::ABL1*^IS^ >10% | Yes | Y253H | – | – | – | – |  |
|  | 1 | MMR or better | No | – | – | – | – | – |  |
| E255K^b^ | 1 | MMR or better | No | – | – | – | – | – |  |
|  | 1 | MMR or better | No | – | – | – | – | – |  |
| E255V^b^ | 1 | MMR or better | No | – | 1 | *BCR::ABL1*^IS^ >10% | Yes | E255V |  |
| F317L^b^ | 1 | *BCR::ABL1*^IS^ >1% to ≤10% | Yes | E355G | 1 | *BCR::ABL1*^IS^ >1% to ≤10% | Yes | F317L |  |
|  | 1 | *BCR::ABL1*^IS^ >10% | Yes | F317L | 1 | MMR or better | No | – |  |
| F359C^b^ | 1 | *BCR::ABL1*^IS^ >10% | Yes | F359C | – | – | – | – |  |
| F359V^b^ | 1 | *BCR::ABL1*^IS^ >1% to ≤10% | Yes | F359V | – | – | – | – |  |
|  | 2 | *BCR::ABL1*^IS^ >10% | Yes | F359V | – | – | – | – |  |
| E459K^c^ | 1 | *BCR::ABL1*^IS^ >10% | Yes | – | – | – | – | – |  |
| W478R^d^ | 1 | MMR or better | No | – | – | – | – | – |  |
| L248V/F317L^b^ | 1 | *BCR::ABL1*^IS^ >0.1% to ≤1% | Yes | F317L | – | – | – | – |  |
| Y253H^b^/F486S^d^ | 1 | *BCR::ABL1*^IS^ >10% | Yes | M244V | – | – | – | – |  |
| M244V^b^ | – | – | – | – | 2 | *BCR::ABL1*^IS^ >10% | Yes | M244V |  |
| Q252H^b^ | – | – | – | – | 1 | *BCR::ABL1*^IS^ >10% | Yes | Q252H |  |
| F359I^b^ | – | – | – | – | 1 | MMR or better | No | – |  |
| R473Q^d^ | – | – | – | – | 1 | MMR or better | Yes | – |  |

MMR, major molecular response (*BCR::ABL1*^IS^ ≤0.1% on the International Scale).

^a^ Mutations were determined by Sanger sequencing. Patients with T315I and V299L *BCR::ABL1* mutations identified at week 1 day 1 were discontinued from study treatment per protocol.

^b^ Adenosine triphosphate–binding region.

^c^ Myristoyl pocket region at a residue that is not in direct contact with asciminib.

^d^ Kinase C-terminal or core regions.
