# Supplementary material for: Asciminib vs bosutinib in chronic-phase chronic myeloid leukemia previously treated with at least two tyrosine kinase inhibitors: longer-term follow-up of ASCEMBL
Source: Leukemia. 2023 Jan 30;37(3):617–26. doi: 10.1038/s41375-023-01829-9 (PMC9991909; doi:10.1038/s41375-023-01829-9)
Supplement: Supplementary file 11 — Table S6 [file 41375_2023_1829_MOESM11_ESM.docx]

**Table S6:** ***BCR::ABL1* mutations at the end of treatment in patients who discontinued treatment**

| **n (%)^a^** | **Asciminib 40 mg twice daily** | **Bosutinib 500 mg once daily** |
| --- | --- | --- |
| **Lack of efficacy or disease progression** | **39** | **30** |
| No mutations detected at end of treatment | 22 (56.4) | 20 (66.7) |
| Missing assessments at end of treatment | 1 (2.6) | 3 (10.0) |
| Mutations detected at end of treatment | 16 (41.0) | 7 (23.3) |
| Newly emerging mutations at end of treatment | 10 (25.6) | 2 (6.7) |
| ATP-binding site | M244V (n=3)^b^  E355G (n=1)^c^  F359V (n=1)  T315I (n=1) | T315I (n=1)  V299L (n=1) |
| Myristoyl pocket | A337T (n=3)  P465S (n=1) | None |
| Mutations at baseline and end of treatment | 6 (15.4) | 5 (16.7) |
| ATP-binding site | F317L (n=2)  F359C/V (n=3)  Y253H (n=1) | M244V (n=2)  E255V (n=1)  F317L (n=1)  Q252H (n=1) |
| **Other^d^** | 33 | 31 |
| Mutations identified at end of treatment | 3 (9.1) | 2 (6.5) |
| Newly emerging mutations | 0 | 0 |
| Mutations at baseline and end of treatment | 3 (9.1) | 2 (6.5) |
| ATP-binding site | F359V (n=1)  T315I (n=2) | T315I (n=1)  V299L (n=1) |
| Myristoyl pocket | E462K (n=1) | None |
| No mutations identified at end of treatment | 16 (48.5) | 14 (45.2) |
| No mutation assessment at end of treatment | 14 (42.4) | 15 (48.4) |

ATP, adenosine triphosphate.
^a^ Patients with T315I and V299L *BCR::ABL1* mutations identified at week 1 day 1 were discontinued from study treatment per protocol.

^b^ One patient had Y253H and F486S *BCR::ABL1* mutations at baseline that were not detected at the time of discontinuation.

^c^ Patient had the F317L *BCR::ABL1* mutation at baseline, which was not detected at the time of discontinuation.

^d^ Includes physician decision, adverse events, patient decision, death, lost to follow-up, and protocol deviation.
