# Supplementary material for: Asciminib vs bosutinib in chronic-phase chronic myeloid leukemia previously treated with at least two tyrosine kinase inhibitors: longer-term follow-up of ASCEMBL
Source: Leukemia. 2023 Jan 30;37(3):617–26. doi: 10.1038/s41375-023-01829-9 (PMC9991909; doi:10.1038/s41375-023-01829-9)
Supplement: Supplementary file 12 — Table S7 [file 41375_2023_1829_MOESM12_ESM.docx]

**Table S7: Laboratory abnormalities**

| **Event, n (%)** | **Asciminib 40 mg twice daily**  **(n=156)** | | **Bosutinib 500 mg once daily**  **(n=76)** | |
| --- | --- | --- | --- | --- |
|  | **All grades** | **Grade 3/4** | **All grades** | **Grade 3/4** |
| **Hematology abnormalities** | | | | |
| Decreased hemoglobin | 56 (36.6) | 3 (2.0) | 41 (53.9) | 4 (5.3) |
| Decreased leukocytes | 69 (45.1) | 14 (9.2) | 22 (28.9) | 4 (5.3) |
| Decreased lymphocytes | 31 (20.4) | 5 (3.3) | 26 (34.2) | 2 (2.6) |
| Decreased neutrophils | 65 (42.8) | 33 (21.7) | 25 (32.9) | 11 (14.5) |
| Decreased platelets | 71 (46.4) | 36 (23.5) | 27 (36.0) | 9 (12.0) |
| Increased prothrombin INR | 14 (9.2) | 1 (0.7) | 8 (11.3) | 1 (1.4) |
| **Selected biochemical abnormalities** | | | | |
| ALT (serum) | 40 (25.6) | 1 (0.6) | 38 (50.0) | 12 (15.8) |
| ALP (serum) | 20 (12.8) | 0 | 9 (11.8) | 0 |
| Amylase (serum) | 20 (12.8) | 2 (1.3) | 10 (13.2) | 0 |
| AST (serum) | 33 (21.2) | 3 (1.9) | 35 (46.1) | 5 (6.6) |
| Bilirubin (serum) | 18 (11.5) | 0 | 3 (3.9) | 0 |
| Cholesterol (serum) | 19 (12.2) | 0 | 6 (7.9) | 0 |
| Creatine kinase | 46 (29.5) | 4 (2.6) | 18 (23.7) | 4 (5.3) |
| Creatinine (plasma/serum) | 24 (15.4) | 0 | 20 (26.3) | 0 |
| Glucose (serum) | 89 (57.1) | 6 (3.8) | 36 (47.4) | 2 (2.6) |
| Pancreatic lipase | 24 (15.4) | 7 (4.5) | 14 (18.4) | 5 (6.6) |
| Triglycerides (plasma/serum) | 69 (44.2) | 8 (5.1) | 23 (30.3) | 2 (2.6) |
| Urate (serum) | 32 (20.5) | 9 (5.8) | 14 (18.4) | 2 (2.6) |

INR, international normalized ratio.
