# Supplementary material for: Asciminib vs bosutinib in chronic-phase chronic myeloid leukemia previously treated with at least two tyrosine kinase inhibitors: longer-term follow-up of ASCEMBL
Source: Leukemia. 2023 Jan 30;37(3):617–26. doi: 10.1038/s41375-023-01829-9 (PMC9991909; doi:10.1038/s41375-023-01829-9)
Supplement: Supplementary file 13 — Table S8 [file 41375_2023_1829_MOESM13_ESM.docx]

**Table S8: Adverse events leading to treatment discontinuation of study treatment by preferred term**

| **Event, n (%)^a^** | **Asciminib 40 mg twice daily**  **(n=156)** | | **Bosutinib 500 mg once daily**  **(n=76)** | |
| --- | --- | --- | --- | --- |
|  | **All grades** | **Grade ≥3** | **All grades** | **Grade ≥3** |
| Number of patients with ≥1 adverse event | 12 (7.7) | 12 (7.7) | 20 (26.3) | 15 (19.7) |
| Thrombocytopenia^b^ | 5 (3.2) | 5 (3.2) | 1 (1.3) | 1 (1.3) |
| Neutropenia^c^ | 4 (2.6) | 4 (2.6) | 3 (3.9) | 3 (3.9) |
| Lipase increased | 3 (1.9) | 3 (1.9) | 0 | 0 |
| Amylase increased | 1 (0.6) | 1 (0.6) | 0 | 0 |
| Cerebral disorder^d^ | 1 (0.6) | 1 (0.6) | 0 | 0 |
| Ejection fraction decreased | 1 (0.6) | 1 (0.6) | 0 | 0 |
| Ischemic stroke | 1 (0.6) | 1 (0.6) | 0 | 0 |
| Alanine aminotransferase increased | 0 | 0 | 4 (5.3) | 3 (3.9) |
| Aspartate aminotransferase increased | 0 | 0 | 2 (2.6) | 1 (1.3) |
| Blood creatinine increased | 0 | 0 | 1 (1.3) | 0 |
| Diarrhea | 0 | 0 | 2 (2.6) | 1 (1.3) |
| Diffuse large B-cell lymphoma | 0 | 0 | 1 (1.3) | 1 (1.3) |
| Drug eruption | 0 | 0 | 1 (1.3) | 0 |
| Hydrothorax | 0 | 0 | 1 (1.3) | 1 (1.3) |
| Pleural effusion | 0 | 0 | 3 (3.9) | 2 (2.6) |
| Pyrexia | 0 | 0 | 1 (1.3) | 1 (1.3) |
| Rash | 0 | 0 | 1 (1.3) | 1 (1.3) |
| Squamous cell carcinoma | 0 | 0 | 1 (1.3) | 1 (1.3) |

^a^ Based on the safety analysis set. Numbers represent counts of patients. A patient with multiple severity grades for an adverse event is only counted under the maximum grade; Medical Dictionary for Regulatory Activities version 24.1, Common Terminology Criteria for Adverse Events version 4.03.

^b^ Includes thrombocytopenia and decreased platelet count.

^c^ Includes neutropenia and decreased neutrophil count.

^d^ Patient was hospitalized as a result of cerebral infarction, with cerebral disorder reported as the final cause of permanent discontinuation.
