# Supplementary material for: Asciminib vs bosutinib in chronic-phase chronic myeloid leukemia previously treated with at least two tyrosine kinase inhibitors: longer-term follow-up of ASCEMBL
Source: Leukemia. 2023 Jan 30;37(3):617–26. doi: 10.1038/s41375-023-01829-9 (PMC9991909; doi:10.1038/s41375-023-01829-9)
Supplement: Supplementary file 14 — Table S9 [file 41375_2023_1829_MOESM14_ESM.docx]

**Table S9: Dose adjustments and discontinuation of study drug**

| **Category^a,b^** | **Asciminib 40 mg twice daily**  **(n=156)** | **Bosutinib 500 mg once daily^c^**  **(n=76)** |
| --- | --- | --- |
| **Patients with dose reduction, n (%)** | | |
| No dose reduction | 91 (58.3) | 41 (53.9) |
| ≥1 dose reduction | 65 (41.7) | 35 (46.1) |
| Only 1 dose reduction | 40 (25.6) | 17 (22.4) |
| 2 dose reductions | 14 (9.0) | 16 (21.1) |
| >2 dose reductions | 11 (7.1) | 2 (2.6) |
| Patients with ≥1 dose reduction by reason, n (%) | | |
| Adverse event | 37 (23.7) | 34 (44.7) |
| Dosing error | 31 (19.9) | 1 (1.3) |
| Physician decision | 2 (1.3) | 2 (2.6) |
| Technical problems | 1 (0.6) | 0 |
| Dispensing error | 0 | 1 (1.3) |
| Median dose reduction per patient (range), n | 1.0 (1-67) | 2.0 (1-3) |
| **Patients with dose interruption, n (%)** | | |
| No dose interruption | 69 (44.2) | 20 (26.3) |
| ≥1 dose interruption | 87 (55.8) | 56 (73.7) |
| Only 1 dose interruption | 40 (25.6) | 26 (34.2) |
| 2 Dose interruptions | 19 (12.2) | 14 (18.4) |
| >2 Dose interruptions | 28 (17.9) | 16 (21.1) |
| Patients with ≥1 dose interruption by reason, n (%) | | |
| Adverse event | 66 (42.3) | 47 (61.8) |
| Dosing error | 21 (13.5) | 13 (17.1) |
| Physician decision | 8 (5.1) | 5 (6.6) |
| Patient decision | 8 (5.1) | 3 (3.9) |
| Dispensing error | 2 (1.3) | 1 (1.3) |
| Technical problems | 1 (0.6) | 0 |
| Median dose interruption per patient (range), n | 2.0 (1-25) | 2.0 (1-11) |
| Median duration of dose interruption (range), days | 27.0 (1-260) | 21.0 (1-128) |
| **Patients with dose increase, n (%)** | | |
| No dose increase | 154 (98.7) | 70 (92.1) |
| ≥1 dose increase | 2 (1.3) | 6 (7.9) |
| Only 1 dose increase | 0 | 6 (7.9) |
| >2 dose increases | 2 (1.3) | 0 |
| Patients with ≥1 dose increase by reason, n (%) | | |
| As per protocol | 0 | 5 (6.6) |
| Dosing error | 2 (1.3) | 1 (1.3) |
| Median dose increase per patient (range), n | 5.5 (4-7) | 1.0 (1-1) |
| Permanent discontinuation, n (%) | 72 (46.2) | 62 (81.6) |

^a^ Based on the safety analysis set.

^b^ Per protocol, adverse events were managed first by dose interruption and upon resolution, dose level was maintained or reduced, or study treatment was permanently discontinued, depending on the type of event and its duration.

^c^ Per protocol, patients randomized to bosutinib still on 500 mg once daily could have their dose escalated to 600 mg once daily if complete hematologic recovery had not been reached by week 8 or complete cytogenetic response by week 12 in patients without grade ≥3 adverse events.
