# Supplementary material for: Asciminib vs bosutinib in chronic-phase chronic myeloid leukemia previously treated with at least two tyrosine kinase inhibitors: longer-term follow-up of ASCEMBL
Source: Leukemia. 2023 Jan 30;37(3):617–26. doi: 10.1038/s41375-023-01829-9 (PMC9991909; doi:10.1038/s41375-023-01829-9)
Supplement: Supplementary file 15 — Table S10 [file 41375_2023_1829_MOESM15_ESM.docx]

**Table S10: Exposure-adjusted incidence rate of non-hematologic adverse events by preferred term (reported in ≥5% of patients in any treatment arm as shown in Table 2)**

| **Exposure-adjusted incidence, n (IR per 100 patient-treatment years)^a^** | **Asciminib 40 mg twice daily**  **(n=156)** | | **Bosutinib 500 mg once daily**  **(n=76)** | |
| --- | --- | --- | --- | --- |
|  | **All grades** | **Grade ≥3** | **All grades** | **Grade ≥3** |
| **Patients with ≥1 event** | **142 (371**.**6)** | **88 (55**.**8)** | **74 (1440**.**8)** | **52 (115**.**8)** |
| Headache | 31 (14.3) | 3 (1.1) | 12 (19.2) | 0 |
| Fatigue | 23 (9.8) | 1 (0.4) | 7 (10.2) | 1 (1.3) |
| Hypertension | 21 (8.3) | 10 (3.8) | 4 (5.3) | 3 (4.0) |
| Arthralgia | 20 (8.4) | 1 (0.4) | 3 (4.1) | 0 |
| Diarrhea | 20 (8.0) | 0 | 55 (234.4) | 8 (11.2) |
| Nausea | 18 (7.4) | 1 (0.4) | 35 (83.3) | 0 |
| Nasopharyngitis | 17 (6.9) | 0 | 3 (4.0) | 0 |
| Abdominal pain | 14 (5.4) | 0 | 12 (18.5) | 1 (1.3) |
| Pain in extremity | 14 (5.5) | 1 (0.4) | 5 (6.8) | 0 |
| Rash | 14 (5.5) | 0 | 18 (31.9) | 3 (4.0) |
| Asthenia | 13 (5.1) | 0 | 1 (1.4) | 0 |
| Cough | 13 (5.0) | 0 | 5 (6.9) | 0 |
| Vomiting | 12 (4.7) | 2 (0.7) | 20 (32.5) | 0 |
| Upper respiratory tract infection | 11 (4.2) | 1 (0.4) | 4 (5.6) | 0 |
| Amylase increased | 9 (3.4) | 1 (0.4) | 4 (5.8) | 0 |
| Aspartate aminotransferase increased | 9 (3.5) | 3 (1.1) | 16 (23.3) | 5 (6.7) |
| Constipation | 8 (3.0) | 0 | 4 (5.3) | 0 |
| Decreased appetite | 8 (3.1) | 0 | 6 (8.0) | 0 |
| Dry skin | 8 (3.0) | 0 | 6 (8.3) | 0 |
| Dyspnea | 8 (3.0) | 0 | 4 (5.4) | 0 |
| Lipase increased | 8 (3.0) | 6 (2.3) | 5 (6.8) | 4 (5.4) |
| Pruritus | 8 (3.1) | 0 | 5 (6.8) | 1 (1.3) |
| Abdominal pain upper | 7 (2.7) | 0 | 5 (6.8) | 1 (1.3) |
| Alanine aminotransferase increased | 7 (2.7) | 1 (0.4) | 23 (36.7) | 11 (15.6) |
| Pyrexia | 6 (2.2) | 2 (0.7) | 6 (8.2) | 1 (1.3) |
| Blood creatinine increased | 5 (1.9) | 0 | 5 (6.9) | 0 |
| Influenza like illness | 3 (1.1) | 0 | 4 (5.6) | 0 |
| Hypophosphatemia | 2 (0.7) | 1 (0.4) | 4 (5.4) | 3 (4.0) |

IR, incidence rate.

^a^ Based on the safety analysis set. The exposure-adjusted IR was calculated by dividing the number of patients with an event by the corresponding sum of the exposure duration for all patients, where duration of exposure in 100 patient-treatment years was counted up to the first qualifying event (or end of time at risk for patients without the event).
