# Supplementary material for: Asciminib vs bosutinib in chronic-phase chronic myeloid leukemia previously treated with at least two tyrosine kinase inhibitors: longer-term follow-up of ASCEMBL
Source: Leukemia. 2023 Jan 30;37(3):617–26. doi: 10.1038/s41375-023-01829-9 (PMC9991909; doi:10.1038/s41375-023-01829-9)
Supplement: Supplementary file 16 — Table S11 [file 41375_2023_1829_MOESM16_ESM.docx]

**Table S11:** **Arterial-occlusive events**

| **Category** | **Asciminib 40 mg twice daily**  **(n=156)** | **Bosutinib 500 mg once daily**  **(n=76)** |
| --- | --- | --- |
| **Patients with AOEs, n (%)** | 8 (5.1) | 1 (1.3) |
| **Patients with events observed by the week 24 cutoff, n (%)** | | |
| Myocardial ischemia | 2 (1.3) | 0 |
| Acute coronary syndrome | 0 | 1 (1.3) |
| Coronary artery disease | 1 (0.6) | 0 |
| Ischemic stroke | 1 (0.6) | 0 |
| Mesenteric artery embolism/thrombosis | 1 (0.6) | 0 |
| **Additional patients with events since the week 24 cutoff, n (%)** | | |
| Cerebral infarction | 1 (0.6) | 0 |
| Myocardial infarction | 1 (0.6) | 0 |
| Troponin increased | 1 (0.6) | 0 |
| **Exposure-adjusted AOE incidence rate (per 100 patient treatment-years)** | | |
| Primary analysis (week 24) | 3.3 | 2.0 |
| Current analysis | 3.0 | 1.4 |

AOE, arterial-occlusive event.
