# Supplementary material for: Asciminib vs bosutinib in chronic-phase chronic myeloid leukemia previously treated with at least two tyrosine kinase inhibitors: longer-term follow-up of ASCEMBL
Source: Leukemia. 2023 Jan 30;37(3):617–26. doi: 10.1038/s41375-023-01829-9 (PMC9991909; doi:10.1038/s41375-023-01829-9)
Supplement: Supplementary file 17 — Table S12 [file 41375_2023_1829_MOESM17_ESM.docx]

**Table S12:** **Characteristics of patients with arterial-occlusive events**

| **Arm** | **Patient** | **Age, years** | **Sex** | **AE term** | **Study day of occurrence** | **CV risk factors at screening** | **Last prior TKIs** |
| --- | --- | --- | --- | --- | --- | --- | --- |
| **Asciminib** | 1 | 71 | F | Myocardial ischemia | 1 | No relevant medical history | Imatinib and nilotinib |
|  | 2 | 52 | F | Myocardial ischemia | 26 | Former smoker, hypertension, low levels of physical activity, unhealthy diet | Nilotinib, dasatinib, imatinib, and ponatinib |
|  | 3 | 65 | M | Ischemic stroke | 56 | Hypertension and left ventricular hypertrophy per ECG | Imatinib, nilotinib, and dasatinib |
|  | 4 | 59 | M | Coronary artery disease | 197 | Hypertension and hyperlipidemia. Chronic cardiac failure reported as part of medical history | Imatinib and nilotinib |
|  | 5 | 57 | M | Myocardial infarction | 253 | No relevant medical history | Imatinib, dasatinib, and nilotinib |
|  | 6 | 62 | F | Mesenteric artery embolism/thrombosis | 260 | Hypertension and low levels of physical activity | Imatinib, dasatinib, and nilotinib. On study day 245 the patient discontinued asciminib and on day 253 started ponatinib |
|  | 7 | 70 | M | Cerebral infarction | 276 | Hypertension and hyperlipidemia | Nilotinib, dasatinib, and ponatinib |
|  | 8 | 63 | M | Troponin increased | 612 | Prior medical conditions included implantable defibrillation insertion. Active conditions included myotonic dystrophy, coronary artery disease, ejection fraction decreased, and hyperlipidemia | Dasatinib and imatinib |
| **Bosutinib** | 1 | 67 | M | Acute coronary syndrome | 173 | Prior myocardial infarction, hypertension, and hyperlipidemia | Imatinib, ponatinib, and nilotinib |

AE, adverse event; CV, cardiovascular; ECG, electrocardiogram; TKI, tyrosine kinase inhibitor.
