# Supplementary material for: Asciminib vs bosutinib in chronic-phase chronic myeloid leukemia previously treated with at least two tyrosine kinase inhibitors: longer-term follow-up of ASCEMBL
Source: Leukemia. 2023 Jan 30;37(3):617–26. doi: 10.1038/s41375-023-01829-9 (PMC9991909; doi:10.1038/s41375-023-01829-9)
Supplement: Supplementary file 18 — CONSORT Checklist [file 41375_2023_1829_MOESM18_ESM.doc]

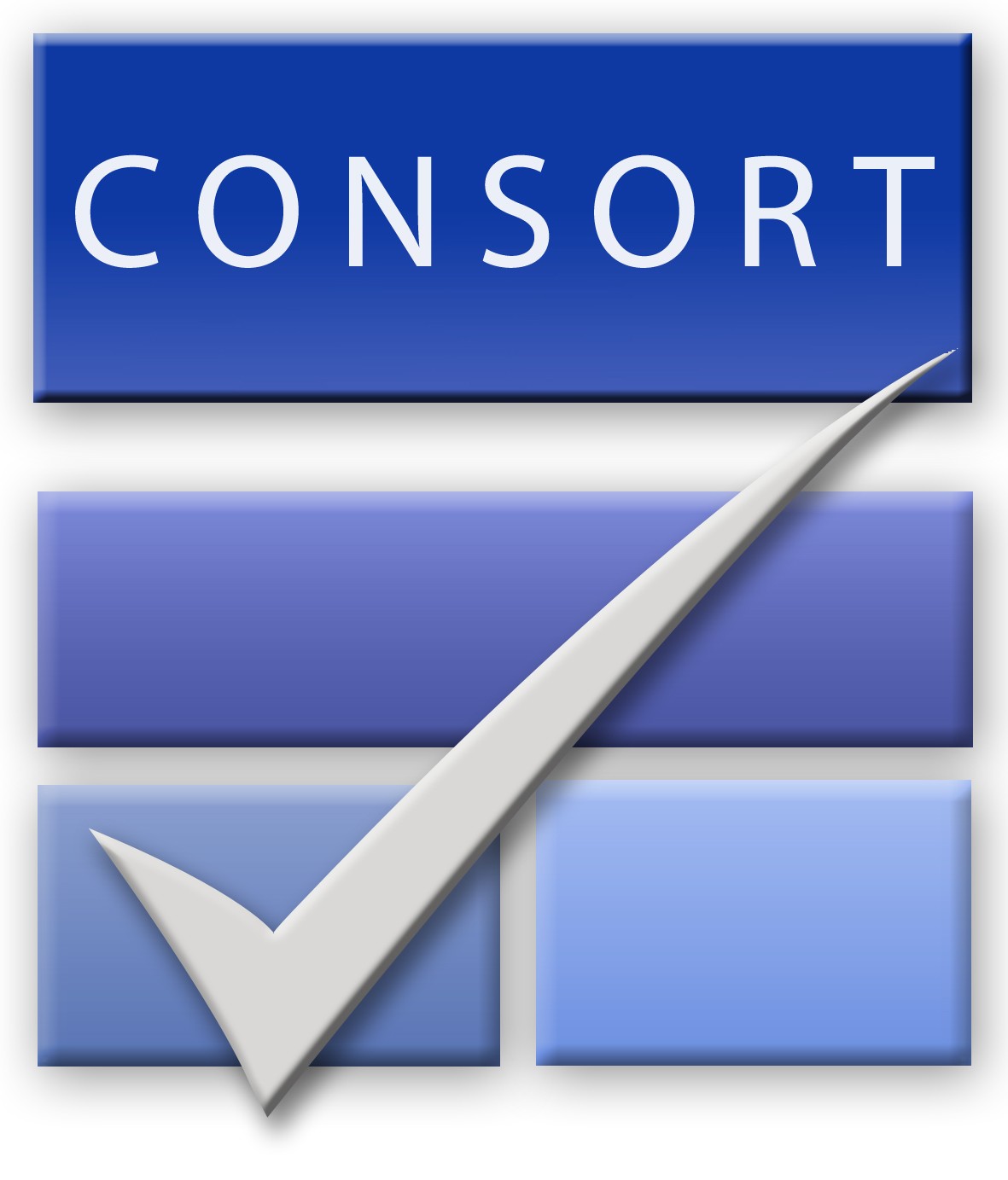
CONSORT 2010 checklist of information to include when reporting a randomised trial*

| Section/Topic | Item No | Checklist item | Reported on page No |
| --- | --- | --- | --- |
| Title and abstract | | | |
|  | 1a | Identification as a randomised trial in the title | Page 1 - Title |
|  | 1b | Structured summary of trial design, methods, results, and conclusions (for specific guidance see CONSORT for abstracts) | Pages 3,4 |
| Introduction | | | |
| Background and objectives | 2a | Scientific background and explanation of rationale | Pages 5,6 |
|  | 2b | Specific objectives or hypotheses | Pages 6,7; Supplemental Appendix, page 3 |
| Methods | | | |
| Trial design | 3a | Description of trial design (such as parallel, factorial) including allocation ratio | Pages 3,6; Supplemental Appendix, page 2; Supplemental Figure S1 |
|  | 3b | Important changes to methods after trial commencement (such as eligibility criteria), with reasons | Page 6; Supplemental Appendix, page 2 |
| Participants | 4a | Eligibility criteria for participants | Pages 4,6; Supplemental Appendix, page 2; Supplemental Figure S1 |
|  | 4b | Settings and locations where the data were collected | Page 6 |
| Interventions | 5 | The interventions for each group with sufficient details to allow replication, including how and when they were actually administered | Pages 4,6,7; Supplemental Appendix, page 2 |
| Outcomes | 6a | Completely defined pre-specified primary and secondary outcome measures, including how and when they were assessed | Pages 4,6,7; Supplemental Appendix, page 3 |
|  | 6b | Any changes to trial outcomes after the trial commenced, with reasons | Not applicable |
| Sample size | 7a | How sample size was determined | Pages 6,7 |
|  | 7b | When applicable, explanation of any interim analyses and stopping guidelines | Not applicable |
| Randomisation: |  |  |  |
| Sequence generation | 8a | Method used to generate the random allocation sequence | Supplemental Appendix page 2 |
|  | 8b | Type of randomisation; details of any restriction (such as blocking and block size) | Not applicable |
| Allocation concealment mechanism | 9 | Mechanism used to implement the random allocation sequence (such as sequentially numbered containers), describing any steps taken to conceal the sequence until interventions were assigned | Supplemental Appendix page 2 |
| Implementation | 10 | Who generated the random allocation sequence, who enrolled participants, and who assigned participants to interventions | Supplemental Appendix page 2 |
| Blinding | 11a | If done, who was blinded after assignment to interventions (for example, participants, care providers, those assessing outcomes) and how | Not applicable |
|  | 11b | If relevant, description of the similarity of interventions | Not applicable |
| Statistical methods | 12a | Statistical methods used to compare groups for primary and secondary outcomes | Page 7; Supplemental Appendix page 3 |
|  | 12b | Methods for additional analyses, such as subgroup analyses and adjusted analyses | Pages 4,6,7; Supplemental Appendix, pages 3-4 |
| Results | | | |
| Participant flow (a diagram is strongly recommended) | 13a | For each group, the numbers of participants who were randomly assigned, received intended treatment, and were analysed for the primary outcome | Pages 7,8 |
|  | 13b | For each group, losses and exclusions after randomisation, together with reasons | Table 1 |
| Recruitment | 14a | Dates defining the periods of recruitment and follow-up | Pages 7,8 |
|  | 14b | Why the trial ended or was stopped | Pages 7,8; Supplemental Figure S1 |
| Baseline data | 15 | A table showing baseline demographic and clinical characteristics for each group | Supplemental Table S2 |
| Numbers analysed | 16 | For each group, number of participants (denominator) included in each analysis and whether the analysis was by original assigned groups | Supplemental Table S1 |
| Outcomes and estimation | 17a | For each primary and secondary outcome, results for each group, and the estimated effect size and its precision (such as 95% confidence interval) | Pages 4,6,7; Supplemental Appendix page 3 |
|  | 17b | For binary outcomes, presentation of both absolute and relative effect sizes is recommended | Not applicable |
| Ancillary analyses | 18 | Results of any other analyses performed, including subgroup analyses and adjusted analyses, distinguishing pre-specified from exploratory | Pages 8,9,10, 12,13; Figures 2,3; Supplemental Tables S3-S6; Supplemental Figures S2-S4 |
| Harms | 19 | All important harms or unintended effects in each group (for specific guidance see CONSORT for harms) | Pages 10,11,12; Figure 4, Tables 2,3; Supplemental Tables S7-S12 |
| Discussion | | | |
| Limitations | 20 | Trial limitations, addressing sources of potential bias, imprecision, and, if relevant, multiplicity of analyses | Pages 13-17 |
| Generalisability | 21 | Generalisability (external validity, applicability) of the trial findings | Pages 13-17 |
| Interpretation | 22 | Interpretation consistent with results, balancing benefits and harms, and considering other relevant evidence | Pages 13-17 |
| Other information | | |  |
| Registration | 23 | Registration number and name of trial registry | Pages 5,6 |
| Protocol | 24 | Where the full trial protocol can be accessed, if available | Not applicable |
| Funding | 25 | Sources of funding and other support (such as supply of drugs), role of funders | Pages 7,19,20 |

*We strongly recommend reading this statement in conjunction with the CONSORT 2010 Explanation and Elaboration for important clarifications on all the items. If relevant, we also recommend reading CONSORT extensions for cluster randomised trials, non-inferiority and equivalence trials, non-pharmacological treatments, herbal interventions, and pragmatic trials. Additional extensions are forthcoming: for those and for up to date references relevant to this checklist, see [www.consort-statement.org](http://www.consort-statement.org/).
